# Integrated analysis reveals effects of bioactive ingredients from *Limonium Sinense* (Girard) Kuntze on hypoxia-inducible factor (HIF) activation

**DOI:** 10.3389/fpls.2022.994036

**Published:** 2022-10-27

**Authors:** Hualong Zhao, Siyuan Wang, Yilu Zhou, Ayse Ertay, Philip T. F. Williamson, Rob M. Ewing, Xinhui Tang, Jialian Wang, Yihua Wang

**Affiliations:** ^1^ School of Marine and Biological Engineering, Yancheng Teachers’ University, Yancheng, China; ^2^ Biological Sciences, Faculty of Environmental and Life Sciences, University of Southampton, Southampton, United Kingdom; ^3^ Institute for Life Sciences, University of Southampton, Southampton, United Kingdom

**Keywords:** *Limonium Sinense* (Girard) Kuntze, cell viability, cell cycle, hypoxia-inducible factor, gallic acid

## Abstract

*Limonium Sinense* (Girard) Kuntze is a traditional Chinese medicinal herb, showing blood replenishment, anti-tumour, anti-hepatitis, and immunomodulation activities amongst others. However, the mechanism of its pharmacological activities remains largely unknown. Here, we investigated the effects of bioactive ingredients from *Limonium Sinense* using an integrated approach. Water extracts from *Limonium Sinense* (LSW) showed a strong growth inhibitory effect on multiple cells in both 2D and 3D cultures. Global transcriptomic profiling and further connectivity map (CMap) analysis identified several similarly acting therapeutic candidates, including Tubulin inhibitors and hypoxia-inducible factor (HIF) modulators. The effect of LSW on the cell cycle was verified with flow cytometry showing a G2/M phase arrest. Integrated analysis suggested a role for gallic acid in mediating HIF activation. Taken together, this study provides novel insights into the bioactive ingredients in *Limonium Sinense*, highlighting the rich natural resource and therapeutic values of herbal plants.

## Introduction

*Limonium Sinense* (Girard) Kuntze is a traditional Chinese medicinal plant belonging to the Plumbaginaceae family and is mainly distributed along seashores and marshes in eastern and southern China, western Taiwan, and Ryukyus Islands (Japan) (Li, 1998; Chaung et al., 2003). Traditionally, the whole plant of *Limonium Sinense* is used for the treatment of fever, hepatitis, hemorrhage, menorrhagia, irregular menstruation, cancer, and other disorders (Dong, 2005).

Multiple bioactive ingredients have been identified from *Limonium Sinense*, including polysaccharides, tannins, alkaloids, flavonoids, terpenes, aliphatic compounds, amino acids, minerals, and vitamins (Xiao ZF et al., 1991, Lin and Chou, 2000; Liu, 2011). It is reported that the major active constituents found in *Limonium Sinense* are flavonoids, including flavanones, flavonols, flavonol glycosides, flavonol glycoside gallates, and flavones (Lin and Chou, 2000; Lin et al., 2000), while polysaccharides are among the most abundant constituents in the roots (Tang et al., 2011). Despite these findings, the mechanism of its pharmacological activities remains to be elucidated. Here we sought to investigate the effects of bioactive ingredients from *Limonium Sinense* using an integrated approach.

## Materials and methods

### Preparation of *Limonium Sinense* water extract

Healthy whole plants of *Limonium Sinense* (Girard) Kuntze were collected from the coastal region in Jiangsu, eastern China (33°09’33.0” N, 120°46’40.4” E). Details for the preparation of LSW are provided in the **Supplementary Methods**.

### Cell culture and reagents

Sources of cell lines and culture conditions were reported earlier (Wang et al., 2014; Liu et al., 2019; Ertay et al., 2020). Details are provided in the **Supplementary Methods**. No mycoplasma contamination was detected in the cell lines used.

### Mammosphere assay and quantifications

Mammosphere assay and quantifications were performed as previously described (Ertay et al., 2020). Details are provided in the **Supplementary Methods**.

### Cell viability assay

Cell viability assay was performed as previously described (Ertay et al., 2020). Details are provided in the **Supplementary Methods**.

### RNA-Seq and bioinformatic analysis

RNA isolation and mRNA sequencing of samples were performed following the manufacturer’s instructions (Novogene, UK) as previously described (Yao et al., 2021; Brereton et al., 2022). Paired-end strategy (2×150) on the Illumina NovaSeq 6000 platform was adopted. The quality control of raw reads, mapping, identification of DEGs, as well as GO term enrichment analysis, KEGG pathway analysis, GSEA, and CMap analysis were performed with details provided in the **Supplementary Methods** and results in **Supplementary Tables S1–5**.

### Flow cytometry

Flow cytometry was performed as previously described (Ertay et al., 2020). Details are provided in the **Supplementary Methods**.

### Western blot analysis

Western blot analysis was performed as previously described (Hill et al., 2019; Wang et al., 2019; Yao et al., 2019; Ertay et al., 2020). Details are provided in the **Supplementary Methods**.

### qRT-PCR

qRT-PCR was performed as previously described (Hill et al., 2019; Wang et al., 2019; Yao et al., 2019; Ertay et al., 2020). Details are provided in the **Supplementary Methods**.

### Immunofluorescence microscopy

Immunofluorescence microscopy was performed as previously described (Ertay et al., 2020). Details are provided in the **Supplementary Methods**.

### Luciferase reporter assay

Luciferase reporter assay was performed as previously described (Yao et al., 2019). Details are provided in the **Supplementary Methods**.

### Integrated data analysis and GSVA score calculation

Gene Expression Omnibus (GEO) datasets on human cells treated with herbal extracts/compounds were screened (**Supplementary Figure 3**), with a summary of datasets in **Supplementary Table S6**. A 15-gene expression signature (*ACOT7*, *ADM*, *ALDOA*, *CDKN3*, *ENO1*, *LDHA*, *MIF*, *MRPS17*, *NDRG1*, *P4HA1*, *PGAM1*, *SLC2A1*, *TPI1*, *TUBB6*, and *VEGFA*), which enables classification of hypoxia-inducible factor (HIF) activity (Buffa et al., 2010; Ye et al., 2019) was used to calculate the HIF score. Details are provided in the **Supplementary Methods**. The herbal extracts or natural compounds that can significantly alter the HIF score are provided in **Supplementary Table S7**.

### High-performance liquid chromatography assay

Details are provided in the **Supplementary Methods**. In brief, chromatography analysis for the identification of gallic acid in LSW was conducted on a Shimadzu^®^ HPLC system (LC-20 AT, SHIMADZU, Japan) equipped with a C18 column (Shim-pack GIS: 5 μm particle size; 4.6 × 250 mm2, P/N: 227-30106-08). Peaks were detected at 271 nm using a UV-Vis detector (SPD-20A), and the peak for gallic acid was identified by comparing the retention time with its standard.

### Statistical analysis

Statistical analyses were performed in GraphPad Prism v7.02 (GraphPad Software Inc, San Diego, CA) unless otherwise indicated as described earlier (Yao et al., 2021; Brereton et al., 2022). Details are provided in the **Supplementary Methods**. Results were considered significant if *P* < 0.05, where *P < 0.05, **P < 0.01, ***P < 0.001, ****P < 0.0001.

## Results

### Bioactive extracts from *Limonium Sinense* show a strong growth inhibitory effect

**Figure 1:**
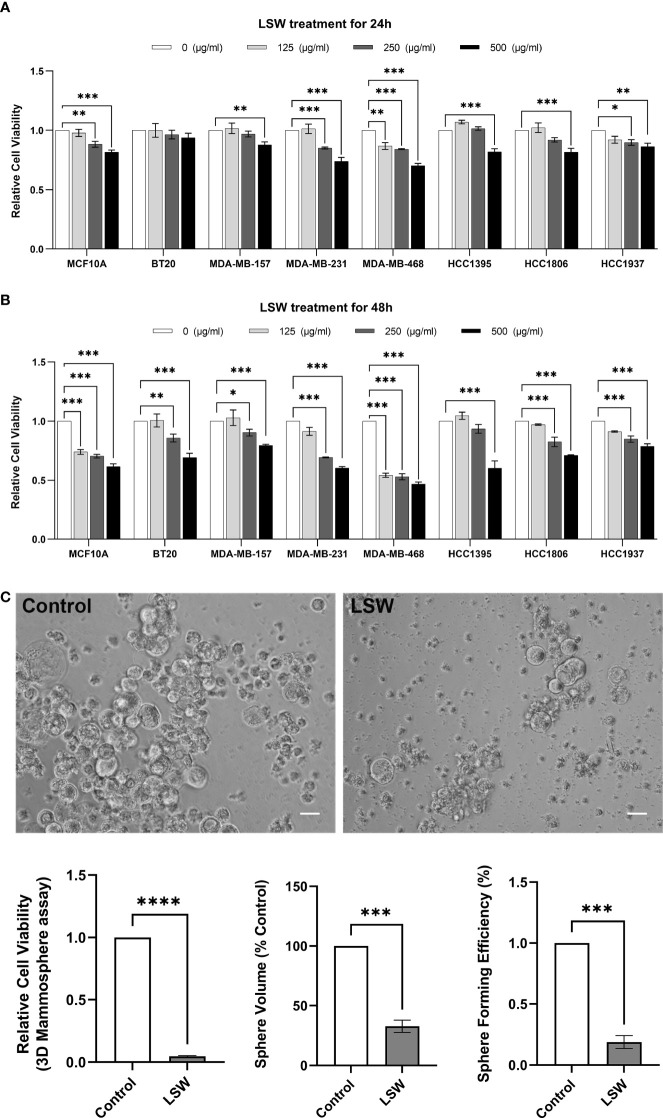
Effects of bioactive extracts from *Limonium Sinense* on cell viability. **(A, B)** Graphs showing relative cell viability in multiple cell lines treated with *Limonium Sinense* water extracts (LSW) at the indicated concentration for 24 **(A)** or 48 hours **(B)** in 2D cultures. Cell-Titer Glo^®^ assay was performed to measure cell viability. Data are mean ± SEM; n = 3 samples per group. ns, not significant; *P < 0.05; **P < 0.01 and ***P < 0.001 by the Two-way ANOVA. **(C)** Representative phase contrast microscopy images showing cell viability (Cell-Titer Glo^®^ assay), sphere volume, and sphere formation efficiency in MDA-MB-468 cells with the indicated treatment cultured in 3D. Scale bar: 50 µm. Data are mean ± SEM. n = 3 samples per group. ***P < 0.001; ****P < 0.0001 by the Student’s *t*-test.

To assess the biological effects of extracts from *Limonium Sinense*, multiple cell lines, including an immortalized human breast epithelial cell line MCF10A and 7 breast cancer cell lines (BT20, MDA-MB-157, MDA-MB-231, MDA-MB-468, HCC1395, HCC1806, and HCC1937), were treated with *Limonium Sinense* water extracts (LSW) followed by a cell viability assay. As shown in **Figures 1A, B**, compared to the control group, the addition of LSW led to a strong inhibition of growth in all the cell lines tested in a dose-dependent manner at 24 (**Figure 1A**) or 48 hours (**Figure 1B**) post-treatment.

It is known that three-dimensional (3D) cell cultures represent their *in vivo* counterparts better than two-dimensional (2D) monolayer cell cultures (Chaicharoenaudomrung et al., 2019). To confirm the effects of LSW on cell viability, a 3D mammosphere assay was performed. Images of spheres were analyzed for sphere formation efficiency and sphere volume, and cell viability was determined using a Cell-Titer Glo^®^ assay. A significant decrease in cell viability (*P* < 0.0001), sphere volume (*P* < 0.001), and sphere formation efficiency (*P* < 0.001) was observed in LSW-treated MDA-MB-468 cells (**Figure 1C**). These experiments showed that bioactive extracts from *Limonium Sinense* exhibit a strong growth inhibitory effect.

### Global transcriptomic changes in MDA-MB-468 cells exposed to bioactive extracts from *Limonium Sinense*

**Figure 2:**
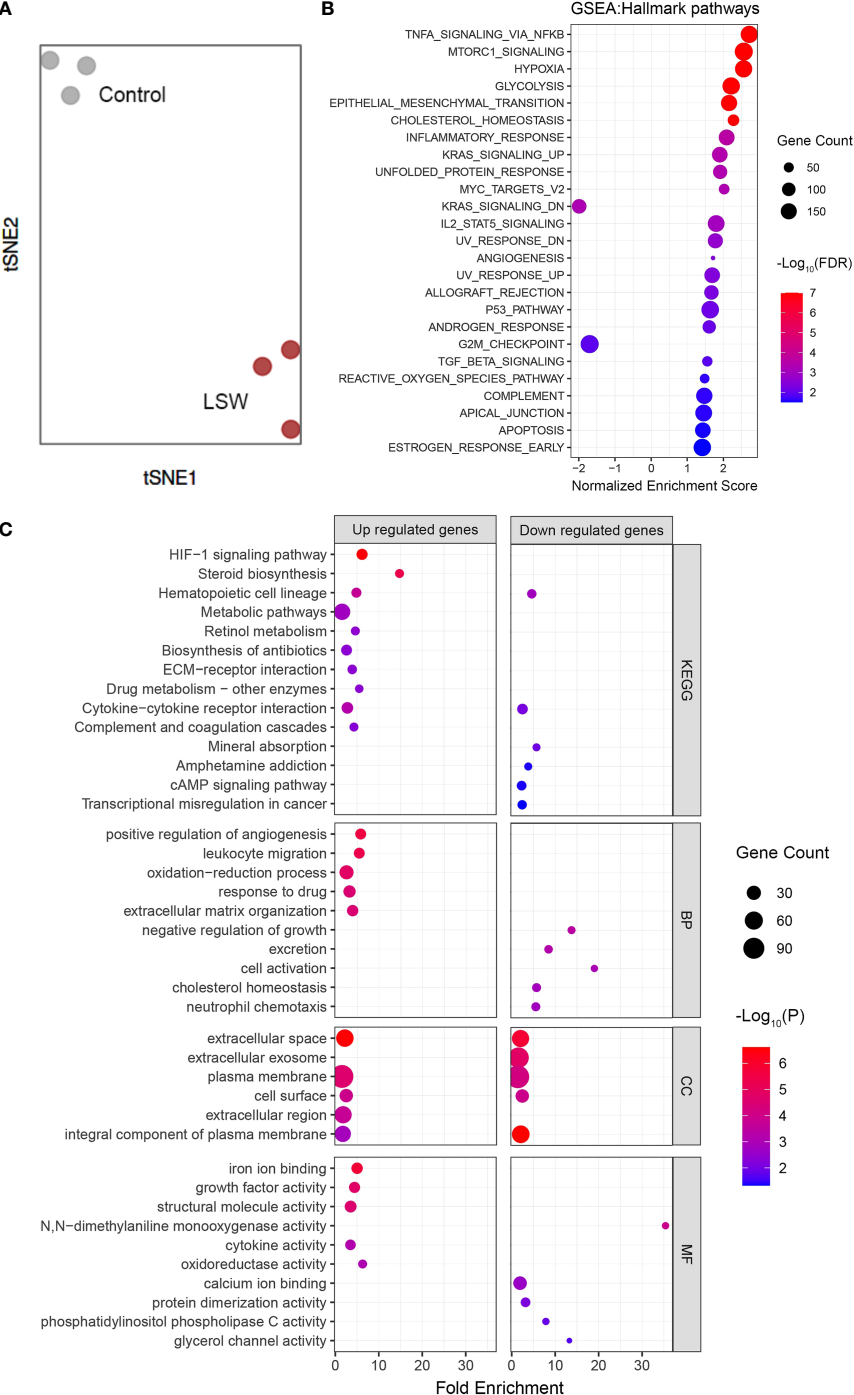
Global transcriptomic changes in MDA-MB-468 cells exposed to bioactive extracts from *Limonium Sinense*. MDA-MB-468 cells were treated with or without *Limonium Sinense* water extracts (LSW, 500 μg/ml) for 48 hours followed by RNA-Seq. **(A)** t-distributed stochastic neighbor embedding (tSNE) plot showing a clear sample separation for control vs. LSW-treated MDA-MB-468 cells (n = 3 samples per group). **(B)** Scatter plot showing Gene Set Enrichment Analysis (GSEA) in MDA-MB-468 cells treated with LSW. The sizes of circles represent gene count, and the colours of circles represent the -Log10 of the false discovery rate (FDR) values. **(C)** Scatter plot showing enriched Kyoto Encyclopedia of Genes and Genomes (KEGG) and Gene Ontology (GO) terms from 3 categories (BP, biological process; CC, cellular component; and MF, molecular function) in MDA-MB-468 cells treated with LSW. The sizes of circles represent gene counts, and the colours of circles represent the -Log_10_ of the *P*-values.

To determine how cells respond to bioactive extracts from *Limonium Sinense*, we characterized the global transcriptomic changes in MDA-MB-468 cells exposed to LSW by performing RNA sequencing (RNA-Seq). Genes with a false discovery rate (FDR) – adjusted *P* value less than 0.05 and ∣Log_2_FoldChange∣ above 1 were considered as differentially expressed genes (DEGs). A total of 987 DEGs were identified, including 456 upregulated and 531 downregulated genes (**Supplementary Figure S1A**; **Supplementary Table S1**). The hierarchical clustering showed that DEGs were grouped into 2 major clusters (**Supplementary Figure S1B**). t-distributed stochastic neighbor embedding (tSNE) analysis showed a clear separation between control and LSW-treated samples (**Figure 2A**).

We then performed pathway enrichment analysis using Gene Set Enrichment Analysis (GSEA) (**Figure 2B**; **Supplementary Table S2**), Kyoto Encyclopedia of Genes and Genomes (KEGG) pathway analysis (**Figure 2C**; **Supplementary Table S3**), and Gene Ontology (GO) enrichment analysis (**Figure 2C**; **Supplementary Table S4**). The GO enrichment analysis was further grouped into the molecular function (MF), biological process (BP), and cellular component (CC) (**Figure 2C**; **Supplementary Table S4**). Interestingly, several disease-related pathological pathways were identified, including G2M_checkpoint (normalized enrichment score, NES = - 1.699; FDR = 0.011) and Hypoxia (NES = 2.558; FDR < 0.0001) in GSEA, and hypoxia-inducible factor (HIF)-1 signaling pathway (*P* < 0.0001) in the KEGG pathway analysis. “Positive regulation of angiogenesis” and “negative regulation of growth” were among the top 10 ranked GO terms from the biological process category (**Figure 2C**). In line with this, GSEA showed enrichment of angiogenesis (**Figure 2B**; **Supplementary Figure S2A**; NES = 1.171; FDR = 0.003). Further analysis showed several angiogenesis-related genes, including *VEGF*, were up-regulated upon LSW treatment (**Supplementary Figure S2B**). These results provide novel insights into the pharmacological activities of bioactive ingredients in *Limonium Sinense*.

### Connectivity Map analysis in MDA-MB-468 cells exposed to bioactive extracts from *Limonium Sinense*

**Figure 3:**
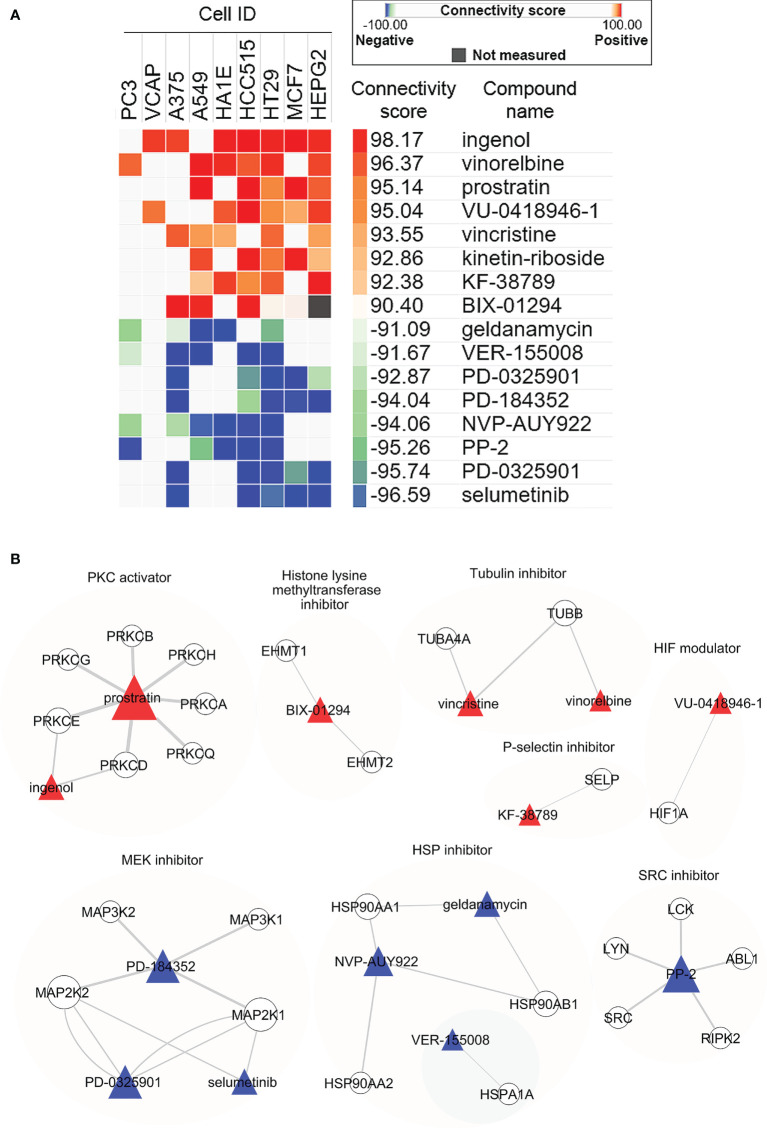
Connectivity Map (CMap) analysis in MDA-MB-468 cells exposed to bioactive extracts from *Limonium Sinense*. **(A)** Heatmap showing the connectivity score for the most significant compounds in 9 cell lines. Cell ID, compound name (Perturbegan), connectivity score (and the colour key), mechanism of action, and target genes for each compound are indicated. Compounds are considered significantly connected with the reference signature when the connectivity score is above 90 (similar) or below -90 (opposite). Compounds are sorted by the decreasing order of their connectivity scores. **(B)** Graph showing the interaction network between compounds and their target genes. The colours and shapes represent the indicated compounds and their target genes. The sizes of the nodes indicated the degrees that the nodes connect to others, and the width of the lines represents the EdgeBetweenness of each gene.

We next employed CMap to explore the small molecular compounds with similar activities to LSW. CMap is a systematic approach that has been applied in pharmacological research to define drug-disease connections (Lamb et al., 2006). CMap analysis reports connectivity between top the 150 up- and down-regulated genes from MDA-MB-468 cells exposed to LSW and 2,837 compounds across 9 cell lines (Subramanian et al., 2017). The connectivity score, which summarizes the connectivity among signatures across cell lines using the median, ranged from + 98.17 to - 96.59 in this analysis (**Supplementary Table S5**). Compounds with connectivity scores above 90 were defined as “positive connectivity”, while compounds with connectivity scores less than -90 were “negative connectivity” (Uva et al., 2021). In total, we identified 8 compounds with positive connectivity and 7 with negative connectivity (**Figure 3A**; **Supplementary Figure S3A**). Among those with positive connectivity, two are Tubulin inhibitors (vinorelbine and vincristine) and 1 HIF modulator (VU-0418946-1). The compound-target network analysis showed distinct clusters among the aforementioned compounds, including Tubulin inhibitors (vinorelbine and vincristine) targeting *TUBB* and HIF modulator (VU-0418946-1) targeting *HIF1A* (**Figure 3B**; **Supplementary Figure S3B**). The CMap analysis supports earlier findings in pathway enrichment analysis showing effects of LSW on G2M_checkpoint in cell cycle and HIF/hypoxia.

### Bioactive extracts from *Limonium Sinense* induce G2/M phase arrest in the cell cycle and HIF activation

**Figure 4:**
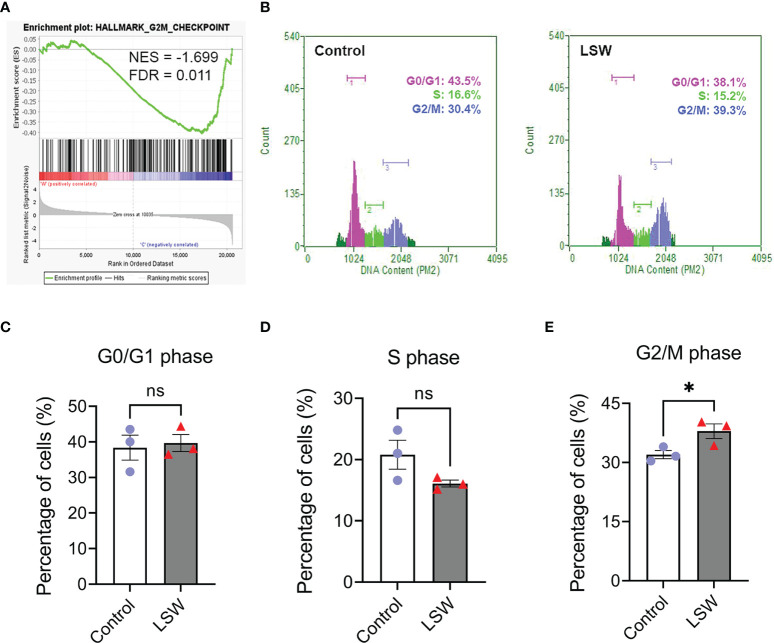
Effects of bioactive extracts from *Limonium Sinense* on cell cycle. **(A)** Gene Set Enrichment Analysis (GSEA) plot showing an enrichment of Hallmark_G2/M_checkpoint in MDA-MB-468 cells treated with *Limonium Sinense* water extracts (LSW, 500 μg/ml). Normalized enrichment score (NES) and false discovery rate (FDR) are indicated. **(B)** Representative flow cytometry histograms of the percentage of cells in G1, S and G2/M phases of the cell cycle from MDA-MB-468 treated with or without LSW (500 μg/ml) for 24 hours. **(C–E)** Graphs showing the percentage of cells in G0/G1 **(C)**, S **(D)**, or G2/M **(E)** phases. Data are mean ± SEM. n = 3 samples per group. ns, not significant; *P < 0.05 by the Student’s *t*-test.

As described above, GSEA identified “Hallmark_G2M_Checkpoint” negatively enriched upon LSW treatment in MDA-MB-468 cells (**Figure 4A**; NES = - 1.699; FDR = 0.011). The effect of LSW on the cell cycle was further verified with flow cytometry analysis (**Figure 4B**), showing a G2/M phase arrest in the cell cycle (**Figure 4E**; *P* < 0.05), with no significant changes in G0/G1 phase (**Figure 4C**) and S phase (**Figure 4D**).

**Figure 5:**
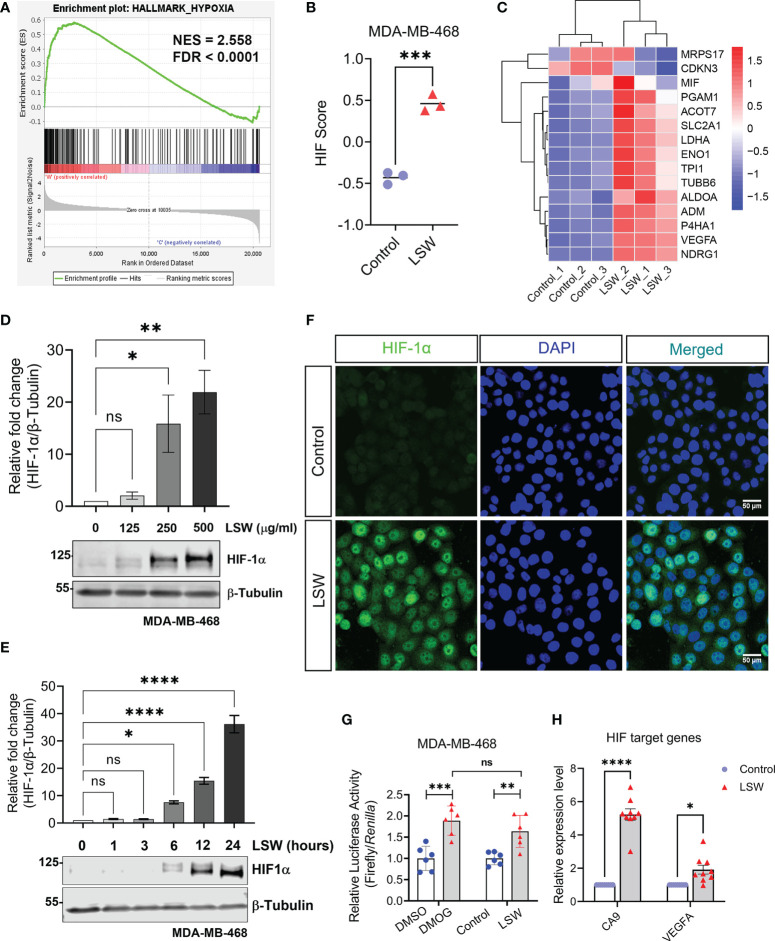
Effects of bioactive extracts from *Limonium Sinense* on hypoxia-inducible factor (HIF) activation. **(A)** Gene Set Enrichment Analysis (GSEA) plot showing enrichment of Hallmark_Hypoxia in MDA-MB-468 cells treated with *Limonium Sinense* water extracts (LSW, 500 μg/ml). Normalized enrichment score (NES) and false discovery rate (FDR) are indicated. **(B)** Graph showing HIF Gene Set Variation Analysis (GSVA) scores from control vs. LSW-treated MDA-MB-468 cells. Data are mean ± SEM; n = 3 samples per group. ***P < 0.001 by the Student’s *t*-test. **(C)** Heatmap showing the expressions of the 15 genes (*ACOT7*, *ADM*, *ALDOA*, *CDKN3*, *ENO1*, *LDHA*, *MIF*, *MRPS17*, *NDRG1*, *P4HA1*, *PGAM1*, *SLC2A1*, *TPI1*, *TUBB6*, and *VEGFA*) used to calculate the HIF score in control vs. LSW-treated MDA-MB-468 cells. Red indicates up-regulation and blue down-regulation. **(D, E)** Protein expressions of HIF-1α in MDA-MB-468 cells with the indicated treatment. β-Tubulin was used as a loading control. Graphs showing relative protein levels of HIF-1α. Data are mean ± SEM; n = 3 samples per group. ns, not significant; *P < 0.05; **P < 0.01; ****P < 0.0001 by One-way ANOVA. **(F)** Immunofluorescence staining of HIF-1α (green) in MDA-MB-468 cells with the indicated treatment. 4’6-Diamidino-2-Pheylindole (DAPI) (blue) was used to stain nuclei. Scale bars: 50 μm. **(G)** Graph showing the Hypoxia Response Element (HRE) reporter assay in MDA-MB-468 cells with the indicated treatment. Values represent the relative fold change of Firefly luciferase to *Renilla* luciferase, normalized against control (1.0). Data are mean ± SEM; n = 6 samples per group. ns, not significant; **P < 0.01; ***P < 0.001 by Dunnett’s multiple comparisons test. **(H)** Graph showing fold change in mRNA levels of HIF-1α target genes (*CA9* and *VEGFA*) in MDA-MB-468 cells with the indicated treatment. β-actin-normalized mRNA levels in control cells were used to set the baseline value at unity. Data are mean ± SEM; n = 9 samples per group. *P < 0.05; ****P < 0.0001 by the Student’s *t*-test.

In addition, our analysis also strongly suggested a role of LSW on HIF activation (**Figures 2**, **3**), including GSEA showing enrichment of “Hallmark_Hypoxia” (**Figure 5A**; NES = 2.558; FDR < 0.0001). To assess the activity of a specific pathway, Gene Set Variation Analysis (GSVA) was used to calculate the score (Hanzelmann et al., 2013). A 15-gene expression signature (*ACOT7*, *ADM*, *ALDOA*, *CDKN3*, *ENO1*, *LDHA*, *MIF*, *MRPS17*, *NDRG1*, *P4HA1*, *PGAM1*, *SLC2A1*, *TPI1*, *TUBB6*, and *VEGFA*), which enables classification of hypoxia-inducible factor (HIF) activity was used to calculate the HIF score (Buffa et al., 2010; Ye et al., 2019). The 15-gene expression signature was derived by selecting genes that were consistently co-expressed upon hypoxia in multiple cancers. A significant increase in the HIF score was observed in LSW-treated samples compared to controls (**Figure 5B**; *P* < 0.001). The expression levels of the 15 genes were visualized in a heatmap with upregulations observed in a majority upon LSW treatment (**Figure 5C**). To validate this finding, the protein level of HIF-1α was measured in MDA-MB-468 cells upon LSW treatment. As shown in **Figures 5D–F**, HIF-1α levels were significantly induced upon LSW treatment in MDA-MB-468 cells in a dose- (**Figure 5D**) and time-dependent manner (**Figure 5E**) as demonstrated by the results from the western blot (**Figures 5D, E**), as well as immunofluorescence staining of HIF-1α (**Figure 5F**). Similar effects on the protein level of HIF-1α were also observed in a human embryonic kidney cell line HEK293T (**Supplementary Figure S4**). Interestingly, the mRNA level of *HIF1A* was not changed, while prolyl hydroxylase domain (PHD) proteins (encoded by *EGLNs*) were not decreased following LSW treatment (**Supplementary Figure S5**), suggesting that LSW treatment induces HIF-1α at the protein level and this is not mediated by down-regulating PHDs.

We then measured the transcriptional activity of HIF using a hypoxia response elements (HRE) reporter system (Masoud and Li, 2015). Upon hypoxia, HIF-α is upregulated and complexed with HIF-1β, binding to the HRE of the gene promoter for transactivation (Kaelin and Ratcliffe, 2008). DMOG (Dimethyloxalylglycine), a non-specific 2-OG analogue that can stabilize and activate HIF (Chan et al., 2016), was used as a positive control. Treatment with DMOG or LSW resulted in a significant increase in the HRE luciferase activity (**Figure 5G**; *P* < 0.001 and *P* < 0.01, respectively). Furthermore, the mRNA levels of HIF target genes, such as *CA9* and *VEGFA*, were also significantly upregulated upon LSW treatment (**Figure 5H**; *P* < 0.0001 and *P* < 0.05, respectively). Taken together, these findings demonstrate that bioactive extracts from *Limonium Sinense* induce G2/M phase arrest of the cell cycle as well as HIF activation.

### Integrated analysis suggests a role for gallic acid within *Limonium Sinense* in mediating HIF activation

**Figure 6:**
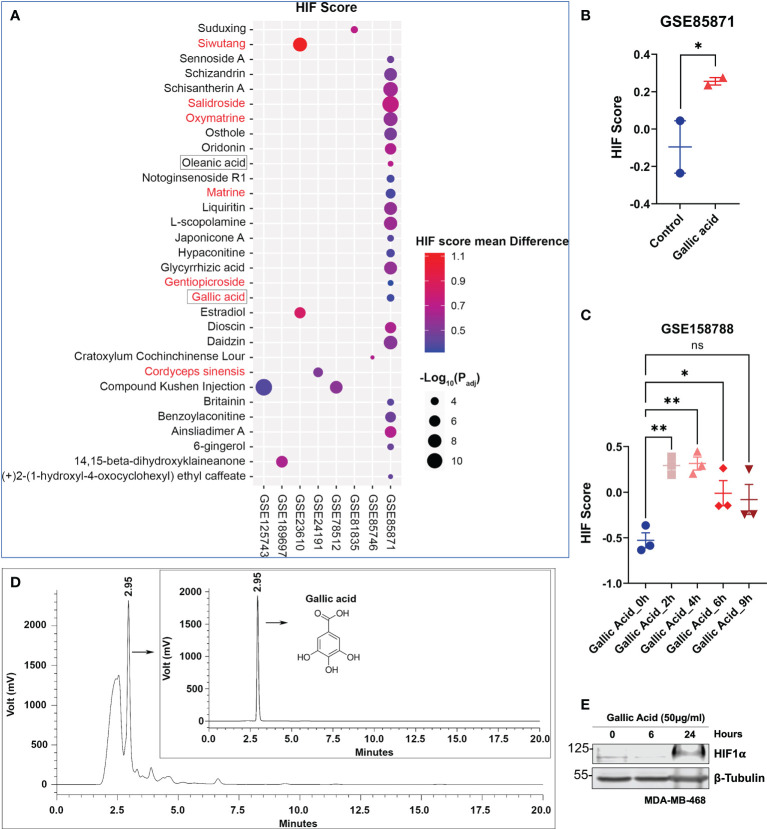
Integrated analysis suggests a role for gallic acid within *Limonium Sinense* in mediating hypoxia-inducible factor (HIF) activation. **(A)** Scatter plot showing natural compounds or herbal extracts that can up-regulate the HIF score. Compounds with red colour are water soluble, and those framed are reported to be present in *Limonium Sinense*. The sizes of circles represent the -Log10 of the *P*_adj_ values, and the colours of circles represent the HIF score mean difference of each compound compared with control samples. **(B, C)** Graphs showing the effect of gallic acid on the HIF score from Gene Expression Omnibus (GEO) dataset GSE85871 **(B)** and GSE158788 **(C)**. Data are mean ± SEM. ns, not significant; *P < 0.05; **P < 0.01 by the Student’s *t*-test **(B)** and one-way ANOVA **(C)**, respectively. **(D)** Chromatograms (HPLC/UV) of *Limonium Sinense* water extracts and gallic acid (insert) in 271 nm. The retention time (2.95 min) and structure for gallica acid are indicated. **(E)** Protein expressions of HIF-1α in MDA-MB-468 cells treated with or without gallic acid (50 μg/ml) for the indicated time. β-Tubulin was used as a loading control.

In order to identify potential bioactive ingredient(s) within *Limonium Sinense* that are responsible for HIF activation, an integrated approach was adopted. We screened Gene Expression Omnibus (GEO) datasets on human cells treated with herbal extracts or natural compounds (**Supplementary Figure S6**). A total of 871 samples were collected from 36 GEO datasets (**Supplementary Table 6**), including 218 control and 653 compound-treated samples in different cells. A HIF score for each sample was calculated using Gene Set Variation Analysis (GSVA) to determine the HIF activity. A total of 31 natural compounds or herbal extracts showed the ability to activate HIF, demonstrated by an increase in the value of the HIF score (**Figure 6A**; **Supplementary Table S7**). Among them, 7 were water soluble and 2 were reported to be present in *Limonium Sinense* (Liu, 2011; Hsu et al., 2015). Five natural compounds or herbal extracts showed an opposite effect on HIF activity (**Supplementary Figure S7**; **Supplementary Table S7**).

The analysis suggested a potential role for gallic acid with *Limonium Sinense* in mediating HIF activation, given the fact that it is water soluble and has been reported to be present in *Limonium Sinense* (Hsu et al., 2015). The HIF score was significantly increased in breast cancer cell line MCF7 (GSE85871; **Figure 6B**) or cervical cancer cell line HeLa (GSE158788; **Figure 6C**) upon gallic acid treatment. The presence of gallic acid in LSW was further confirmed by using the high-performance liquid chromatography (HPLC) analysis, showing the peak of gallic acid in LSW and its standard with a retention time of 2.95 min (**Figure 6D**). The concentration of gallic acid in LSW is 2.24 μg/ml. To validate the effect of gallic acid on HIF, the protein level of HIF-1α was measured in MDA-MB-468 cells following gallic acid treatment. As shown in **Figure 6E**, the HIF-1α protein level was induced upon treatment in MDA-MB-468 cells in a time-dependent manner. Together, these results suggest a role for gallic acid within *Limonium Sinense* in mediating HIF activation, at least partially.

## Discussion and conclusions

*Limonium Sinense* used in traditional Chinese medicine is often extracted with boiling water to make an aqueous extract for oral uptake (Chaung et al., 2003). Water extracts from *Limonium Sinense* (LSW) show antiviral, antitumour, and immunomodulatory activities in previous studies (Kuo et al., 2002; Tang et al., 2008; Tang et al., 2012; Tang et al., 2014; Hsu et al., 2015). In this study, we were able to show that LSW treatment leads to a strong inhibition of growth, potentially by arresting the cell cycle at the G2/M phase. CMap analysis identified Tubulin inhibitors as similarly acting therapeutic candidates in LSW. Tubulin inhibitors are chemotherapy drugs that interfere directly with the tubulin system that enables a cell to undergo mitosis (Janke and Magiera, 2020). Among the various mechanisms of action of natural compounds or herbal extracts, their ability to interact with Tubulin is one of the most important (Kingston, 2009). Whether the effect of *Limonium Sinense* on the cell cycle is *via* its interaction with Tubulin merits further investigation.

The name of *Limonium Sinense* in Chinese means “an herb that can increase blood count”. It is often used to replenish blood in the body, and also for the treatment of hemostasis, anemia, menorrhagia, irregular menstruation, and blood collapse. However, the reason why *Limonium Sinense* possesses such an outstanding blood-enriching function along with its mechanism of action is largely unknown. The production of red blood cells relies predominantly on the cytokine erythropoietin (EPO) and its transcription factor HIF (Lee and Percy, 2011). Studies have shown that regulating HIF levels provide novel therapeutic strategies for a broad variety of diseases, including anemia (De Bels et al., 2011; Semenza, 2012; Schodel and Ratcliffe, 2019). In our analysis, GSEA showed enrichment of angiogenesis. In addition, “positive regulation of angiogenesis” was the top-ranked GO term in the biological process category. Further analysis showed several angiogenesis-related genes, including *VEGF*, were up-regulated upon LSW treatment. We found LSW treatment induces a strong HIF activation. The integrated analysis identified gallic acid as a potentially bioactive ingredient within *Limonium Sinense* mediating this effect, although further experiments are needed to investigate whether the biological effect of LSW is mainly mediated by gallic acid.

Hypoxia plays a crucial role at both cellular and physiological levels in all animals (Wang et al., 2018) and is one of the characteristic pathophysiological features of many common disorders (Semenza, 2019). HIF-α is the most important regulator of cellular responses to hypoxia (Darby and Hewitson, 2016). Under normoxia, the protein level of HIF-α is low due to the oxygen-dependent hydroxylation by members of the prolyl hydroxylase domain (PHD) family (Myllyharju, 2013). This allows HIF-α to interact with tumor suppressor von Hippel-Lindau (pVHL), thereby leading to polyubiquitylation and degradation (Kaelin and Ratcliffe, 2008). When the oxygen level is low/deprived (hypoxia), or cells lack a functional pVHL, HIF-α accumulates and dimerizes with HIF-1β, translocates to the nucleus, and activates the transcription of multiple genes involved in erythropoiesis, angiogenesis and energy metabolism (Kaelin and Ratcliffe, 2008). The genes targeted by HIF make it an appealing pharmacological target, especially for the treatment of diseases including anaemia, ischaemic stroke, and wound healing *via* PHD inhibition-mediated upregulation of HIF (Davis et al., 2018). For example, PHD inhibitors FG4592 (Roxadustat, FibroGen), GSK1278863 (Daprodustat, GlaxoSmithKline), Bay85-3934 (Molidustat, Bayer), and AKB-6548 (Vadadustat, Akebia) are currently in clinical use or trials for anaemia treatment in patients with chronic kidney disease (Chan et al., 2016; Yeh et al., 2017). In our analysis, we found the mRNA level of *HIF1A* was not changed, while PHDs were not decreased by LSW treatment. These results suggest that LSW treatment induces HIF-1α at the protein level and this is not mediated by down-regulating PHDs. In line with our findings, Tsukiyama and colleagues reported that gallate can inhibit PHD activity, thereby reducing the HIF degradation rate and increasing the protein level of HIF-1α (Tsukiyama et al., 2006). The gallate binds to the active site of PHD with its phenolate oxygen atoms chelating Fe^2+^ and the carboxyl group binding to Arg383.

Taken together, this study provides novel insights into the bioactive ingredients in *Limonium Sinense*, highlighting the rich natural resource and therapeutic values of herbal plants. However, given its potential to generate many other effects, consideration is required when *Limonium Sinense* is used clinically.

## Data availability statement

The original contributions presented in the study are publicly available. This data can be found here: Gene Expression Omnibus (GEO)-NCBI, GSE205252.

## Author contributions

YW and JW designed the study. HZ, SW, YZ, and AE performed the experiments. YW, JW, HZ, SW, and YZ analyzed the data and finalized the figures, and all other authors contributed to the data interpretation. YW, JW, HZ, PW, RE, and XT drafted the manuscript with input from other authors. All authors read and approved the final manuscript.

## Acknowledgments

This project was funded in part by Yancheng Teachers’ University and the University of Southampton. YW was supported by the Medical Research Council [grant no: MR/S025480/1] and an Academy of Medical Sciences/the Wellcome Trust Springboard Award [grant no: SBF002\1038]. SW was supported by China Scholarship Council. YZ was supported by an Institute for Life Sciences Ph.D. Studentship. AE was supported by the Wessex Medical Trust. The authors gratefully acknowledge the Imaging and Microscopy Centre at the Biological Sciences (The University of Southampton) for their support and assistance in this work. We would also like to thank Prof Christopher Schofield (The University of Oxford) and Dr. Matthias Baud (The University of Southampton) for helpful discussions and advice. For the purpose of open access, the authors have applied a CC-BY public copyright license to any Author Accepted Manuscript version arising from this submission.

## Conflict of interest

The authors declare that the research was conducted in the absence of any commercial or financial relationships that could be construed as a potential conflict of interest.

## Publisher’s note

All claims expressed in this article are solely those of the authors and do not necessarily represent those of their affiliated organizations, or those of the publisher, the editors and the reviewers. Any product that may be evaluated in this article, or claim that may be made by its manufacturer, is not guaranteed or endorsed by the publisher.

## Abbreviations

BP, biological process; CC, cellular component; CMap, connectivity map; DEG, differentially expressed gene; EPO, erythropoietin; FDR, false discovery rate; GEO, gene expression omnibus; GO, gene ontology; GSEA, gene set enrichment analysis; GSVA, gene set variation analysis; HIF, hypoxia-inducible factor; HPLC, high-performance liquid chromatography; HRE, hypoxia response elements; KEGG, kyoto encyclopedia of genes and genomes; LSW, *Limonium Sinense* water extract; MF, molecular function; NES, normalized enrichment score; PHD, prolyl hydroxylase domain; RNA-Seq, RNA sequencing; tSNE, t-distributed stochastic neighbour embedding; VHL, von Hippel-Lindau.
