# Supplementary material for: Integrated analysis reveals effects of bioactive ingredients from Limonium Sinense (Girard) Kuntze on hypoxia-inducible factor (HIF) activation
Source: Front Plant Sci. 2022 Oct 27;13:994036. doi: 10.3389/fpls.2022.994036 (PMC9646520; doi:10.3389/fpls.2022.994036)
Supplement: Supplementary file 1 [file DataSheet_1.zip › Additional File_submitted/Supplementary materials_submitted.pdf]

Integrated analysis reveals effects of bioactive ingredients from *Limonium Sinense* (Girard) Kuntze on hypoxia-inducible factor (HIF) activation

Supplementary Materials

Table of contents

Supplementary Methods 3

1. Preparation of *Limonium Sinense* water extract (LSW) 3

2. Cell culture and reagents 3

3. Mammosphere assay and quantifications 4

4. Cell viability assay 4

5. RNA-seq and bioinformatic analysis 4

6. Flow cytometry 6

7. Western blot analysis 6

8. Real-time qPCR analysis 6

9. Immunofluorescence microscopy 7

10. Luciferase reporter assay 8

11. Integrated data analysis and Gene Set Variation Analysis (GSVA) score calculation 8

12. High-performance liquid chromatography (HPLC) assay 9

13. Statistical analysis 10

Supplementary Figures 11

Supplementary Figure 1 11

Supplementary Figure 2 12

Supplementary Figure 3 13

Supplementary Figure 4 14

Supplementary Figure 5 15

Supplementary Figure 6 16
Supplementary Figure 7 17
Supplementary Tables *18*
R Scripts *19*
1. R codes for DEG analysis in GSE205252 19
2. R codes for Figure 2B 21
3. R codes for Figure 2C 22
4. R codes for Figure 5B 23
5. R codes for Figure 5C 24
6. R codes for Figure 6A 25
7. R codes for Figure 6B 26
8. R codes for Figure 6C 27
9. R codes for Supplementary Figure 1A 28
10. R codes for Supplementary Figure 1B 29
11. R codes for Supplementary Figure 2B 30
12. R codes for Supplementary Figure 7 31
References *32*
Raw data for 3D culture *34*
Raw data for cell cycle *35*
Raw data for western blot *37*
Raw data for immunofluorescence assay *38*
Raw data for HPLC *39*

Supplementary Methods

1. Preparation of *Limonium Sinense* water extract (LSW)

Healthy whole plants of *Limonium Sinense* (Girard) Kuntze were collected from the coastal region in Jiangsu, eastern China (33°09'33.0" N, 120°46'40.4" E). The whole plants were washed, and oven-dried at 60 °C until the weight was constant. Dried plants were crushed and extracted with distilled water at 95 °C for 2 hours at 1:200 (m/v) ratio. The extract process was repeated 3 times under the same conditions. The extracts were then put to suction filtration, rotary evaporated, and freeze-dried to powder. The powder was aliquoted and stored at - 20 °C until future use when extracts were diluted with sterile water and filtered through a 0.45 μm filter (Millipore filter membranes, Merck, UK).

2. Cell culture and reagents

Sources of cell lines and culture conditions were reported earlier (Wang, Bu et al. 2014, Liu, Ertay et al. 2019). Human breast cancer cell lines (HCC1806, BT20, MDA-MB-157, MDA-MB-231, MDA-MB-468, HCC1395, HCC1937) and an immortalized human breast epithelial cell line MCF10A were obtained as NCI-ICBP45 kit procured through American Type Culture Collection (ATCC) (ATCC Breast Cancer Cell Panel, Manassas, VA, USA). Cell lines were authenticated by ATCC using short tandem repeat DNA profiling, and each cell culture was examined by light microscopy and compared with images published by ATCC and the Integrative Cancer Biology Program (ICBP; http://icbp.lbl.gov/breastcancer/celllines.php) to verify identity (Weigelt, Warne et al. 2011). HCC1806, HCC1395 and HCC1937 cells were maintained in Roswell Park Memorial Institute (RPMI) 1640 medium, (Gibco® by Life Technology) with 10% fetal bovine serum (FBS) and 1% (v/v) penicillin/streptomycin, (Gibco® by Life Technology). BT20, MDA-MB-157, MDA-MB-231 and MDA-MB-468 cell lines were maintained in Dulbecco's modified Eagle's medium (DMEM) (Gibco® by Life Technology) with 10% FBS and 1% (v/v) penicillin/streptomycin. MCF10A cells were maintained in a 1:1 mixture of DMEM and Ham's F12 medium supplemented with 5% horse serum (Invitrogen), 20 ng/ml EGF, 100 ng/ml cholera

toxin, 10 μg/ml insulin, 500 ng/ml hydrocortisone and antibiotics. All cells were kept at 37 °C and 5% CO_2. No mycoplasma contamination was detected in the cell lines used. Gallic acid was purchased from Thermo Scientific™.

3. Mammosphere assay and quantifications

3D cultures were performed as previously described (Ertay, Liu et al. 2020). Cells were cultured in 96-well ultralow attachment plate in 100 μl at plating densities between 3,000 and 7,000 cells/well. Cells were cultured in 1:1 DMEM:F12, (Gibco® by Life Technology) media plus 1% P/S, 2% B27 (Gibco® by Life Technology), 20 ng/ml epidermal growth factor (EGF), (PEPROTECH) and 20 ng/ml basic fibroblast growth factor (bFGF) (PEPROTECH) at 37 °C and 5% CO_2 for 14 days. After the incubation period, the images were taken using with × 40 magnification. The mammospheres that were equal to or greater than 50 μm in diameter were counted to calculate the mammosphere formation efficiency (MFE) with the following equation: (# of mammospheres per well)/(# of cells seeded per well) × 100. Additionally, the volumes of the mammospheres were also calculated using the formula of Volume = $(4/3)\pi r^3$. ImageJ (version1.52a) was used to determine the MFE and volume of sphere.

4. Cell viability assay

Cell viability assay was performed as previously described (Ertay, Liu et al. 2020). Cells were plated into 96-well plate with a density of 8000 cells/well. CellTiter-Glo® Luminescent cell viability assay (Promega) was performed 24h after treatment according to the manufacturer's protocol using GloMax® Discover Microplate Reader (Promega). For cell viability in 3D cultures, 100 μl of CellTiter-Glo® reagent was added into each well and incubated at room temperature for 1h, followed by measurement.

5. RNA-seq and bioinformatic analysis

RNA isolation and mRNA sequencing of samples were performed following the manufacturer's instructions (Novogene, UK) as previously described (Yao, Zhou et al. 2021, Brereton, Yao et al. 2022). The MDA-MB-468 cells were treated with LSW for 48h. Total RNA was isolated using RNeasy mini kit (Qiagen) according to

manufacturer's instructions and quantified using a Nanodrop Spectophotometer 2000c (Thermo Fisher Scientific). A total amount of 3 μg RNA per sample was used as input material for library construction. Sequencing libraries were generated using NEBNext® UltraTM RNA Library Prep Kit for Illumina® (NEB, Ipswich, Massachusetts, USA) following manufacturer's instruction. Libraries were pooled in equimolar and sequenced using the paired-end strategy (2 × 150) on the Illumina NovaSeq 6000 platform following the standard protocols (Novogene, UK). Raw read counts were imported into RStudio (version 4.2.0) and analyzed by using R packages. Transcripts with low abundance (under 10 counts across all samples) were removed. The R codes were provided in the R Scripts in the supplementary materials. Genes with $|Log_2FoldChange|$ above 1 and *P* values adjusted by using Benjamini-Hochberg (BH) method less than 0.05 were considered as differentially expressed genes (DEGs). The details of DEGs from LSW *vs*. Control are shown in Supplementary Table 1.

The t-distributed stochastic neighbor embedding (t-SNE) plot was generated from Omics Playground which is an online self-service analytics platform (https://bigomics.ch/omics-playground/).

The collection of hallmark gene sets generated from the GSEA software (version 4.1.0) (with registration) (Mootha, Lindgren et al. 2003, Subramanian, Tamayo et al. 2005). Gene ontology (GO) terms and Kyoto Encyclopedia of Genes and Genomes (KEGG) enrichment analysis of DEGs were generated through DAVID website tools (https://david.ncifcrf.gov) with default parameters. *P* values adjusted by Benjamini-Hochberg (BH) method were used to estimate the statistical significance, $P < 0.05$ was defined as significant for KEGG, GO enrichment and GSEA, respectively. The significant GSEA hallmark pathways data from LSW vs Control are provided in Supplementary Table 2, KEGG enrichment analysis in Supplementary Table 3 and GO enrichment analysis in Supplementary Table 4.

A public online tool Connectivity Map (CMap) (http://clue.io/) (with registration) was performed to determine which target drugs might have similar or opposite expression signatures with LSW, the mechanism of actions (MOA) and drug-target were investigated as well. Top 150 up regulated genes and top 150 down regulated genes were submitted in the CMap Query tool in December 2021, and compounds with connectivity score above 90 were considered as positive connectivity, whereas

compounds with connectivity score less than -90 were considered as negative connectivity. The summary data of positive and negative connectivity compounds is provided in Supplementary Table 5.

6. Flow cytometry

Flow cytometry was performed as previously described (Ertay, Liu et al. 2020). After treated with LSW for 24 hours, cells were fixed with 70% ethanol and kept at 4 °C for up to 2 weeks. Then, cells were treated with 0.25% Triton-X-100, 200 µg/ml RNAse A and 50 µg/ml propidium iodide (PI), and analyzed by FACS, Guava.

7. Western blot analysis

Western blot analysis was performed with lysates from cells lysed with urea buffer (8 M urea, 1 M thiourea, 0.5% CHAPS, 50 mM 1,4-Dithiothreitol (DTT) and 24 mM spermine) (Hill, Li et al. 2019, Wang, Xiong et al. 2019, Yao, Conforti et al. 2019, Ertay, Liu et al. 2020). Primary antibodies were from Cell Signalling Technology (β-Tubulin, Cat No: 86298; 1:1000) and BD Transduction Laboratories™ (HIF-1α, Cat No: 610959; 1:1000). Signals were detected using an Odyssey imaging system (LI-COR) and evaluated by ImageJ (version1.52a) software (National Institutes of Health) (Berhesda, MD, USA).

8. Real-time qPCR analysis

RNA extraction was performed by RNeasy® Mini Kit (Qiagen) manufacturer's protocol and Nanodrop Spectrophometer 2000c (Thermo Fisher Scientific) was used to quantify RNA concentration. QuantiNova™ SYBR Green RT-PCR kits (Qiagen) were used with *CA9* (QT00011697), *VEGFA* (QT01010184) and *ACTB* (β-actin, QT00095431) gene specific primers (QuantiTect Primer Assays, Qiagen). Relative mRNA levels of target genes were normalized to *ACTB* (β-actin).

9. Immunofluorescence microscopy

The immunofluorescence assay was performed as previously described (Ertay, Liu et al. 2020). When the cells reached 80 - 90% confluency, media were removed and cells were gently washed with 1× PBS twice. One ml 4% paraformaldehyde (PFA) (Thermo Fisher Scientific, UK) in 1× PBS was added to fix the cells for 15 minutes. PFA was removed and cells were washed with 1× PBS. For permeabilisation of cells, 500 µl of 0.1% TironX-100 (Thermo Fisher Scientific, UK) in 1× PBS was added to the each well of 12 well plate and the slide was transferred from 6 well plate into 12 well plate and incubated in 0.1% TironX-100 for 5 minutes on ice. This was followed by washing the slides with 1× PBS twice. Then, cells on the slides were blocked in 0.2% Fish Skin Gelatine (Sigma Aldrich, UK) in 1× PBS for 60 minutes at room temperature. Meanwhile, anti-HIF-1α primary antibody was prepared in blocking buffer with 1:50 dilution and paraflim was put on the foilwrapped container. To moisture the container, wet tissues were put into the side of box. Forty-five µl of primary antibody (BD Transduction Laboratories™, HIF-1α, Cat No: 610959; 1:100) was put on the paraflim and the excess buffer was got rid of from the slides and slides were put on the antibody upside down for 60 minutes at room temperature. 60 minutes after the primary antibody incubation, slides were flipped and put into 12 well plate and washed with 1× PBS 3 times, each time for 15 minutes on the rocker. Then, secondary antibody with 4'6-Diamidino-2-Pheylindole (DAPI) (Invitrogen, UK) was prepared in 1× PBS with the dilution of 1:400 and 1:1000, respectively. New paraflim was put into the box, 95 µl of secondary antibody was put onto paraflim, and slides were put onto paraflim upside down and incubated at room temperature for 60 minutes. Slides were washed with 1× PBS as previously by avoiding light. 8 µl of mounting solution was added to the cover slip and slide was put on the cover slip upside down and left to air dry overnight by avoiding light. Protein expression was detected using Alexa Fluor (1:400, Molecular Probes) for 20 minutes. Immunostained cells were analyzed and photographed using an Olympus IX83 inverted fluorescence microscope.

10. Luciferase reporter assay

The luciferase reporter assay was performed as previously described (Yao, Conforti et al. 2019). Cells were transfected using Lipofectamine 3000 (Life technology) in a 96-well plate with 100 ng of *Renilla* along with 100 ng of HRE reporter per well. Cells were washed with 1 × PBS and lysed by trypsin (0.05% trypsin, Gibco), then centrifuged at 500 g for 5 minutes. Cell pellet was then re-suspended in certain amount of complete media before plating on a 96 well plates (Usually 100 μl of medium for each well) at 70%-80% confluency. For each well to be transfected, 0.1 μl of Lipofectamine 3000 reagent (Life technology) was diluted in 5 μl of Opti-MEM medium. Mixed well reagent was made and short vortexed. Diluted plasmids and Lipofectamine 3000 were mixed by pipetting up and down and the lipid-DNA mixture was incubated at room temperature for 15 minutes. Cells were transfected at 37°C for 48 hours before analysis. Then cells were treated with LSW for 24 hours. The transcriptional assay was carried out using the Dual-Luciferase reporter assay system (Promega, UK) following the manufacturer's protocol. Transfected cells from LSW treatment were wash with 1 × PBS prior to lysis in a 96-well plate. Cells were lysed in 100 μl of passive lysis buffer and put on a room temperature shaker for 15 minutes. Freezing lysates at -20 °C facilitated the lysis. Five μl of lysate was analyzed for each well in a 96 well white plate. Triplets were used for each transfection and 25 μl LAR II was first added and mixed by pipetting to measure the firefly luciferase activity. Another 25 μl of stop and go reagent was then added to help identify the *Renilla* activity. The final Dual-Luciferase Reporter activity was normalized based on both measurements.

11. Integrated data analysis and Gene Set Variation Analysis (GSVA) score calculation

We screened Gene Expression Omnibus (GEO) datasets on human cells treated with herbal extracts/compounds (Supplementary Fig. 6), by searching the keywords

"(Traditional Chinese Medicine) AND (herb) AND (herbal) AND (medicinal) AND (medicinal plant)" and publication dates before 18/01/2022 in the National Center for Biotechnology Information (NCBI) GEO platform. Initially, 673 datasets were identified. Then we only included datasets that met the following criteria: 1) mRNA expression data; 2) human sapiens samples; 3) natural products derived from medicinal plants; 4) minimum 2 biological replicates. Duplicate datasets were removed. Datasets detected less than 10,000 genes were excluded to balance the number of analyzed genes and sample size. Microarray probe IDs were translated to gene symbols according to the GPL annotation files provided in the GEO database. Probes mapped to multiple gene symbols were removed and genes mapped to multiple probe IDs were summarized by calculating the mean. Expression data of the same conditions from multiple datasets were integrated. Only genes that are present across all the platforms remained for further analysis. A total of 36 datasets including 873 samples were collected in the data merging analysis. The details for datasets included are shown in Supplementary Table 6, and list of compounds that can significantly alter the HIF score in Supplementary Table 7.

A 15-gene expression signature (*ACOT7*, *ADM*, *ALDOA*, *CDKN3*, *ENO1*, *LDHA*, *MIF*, *MRPS17*, *NDRG1*, *P4HA1*, *PGAM1*, *SLC2A1*, *TPI1*, *TUBB6* and *VEGFA*), which enables classification of hypoxia-inducible factor (HIF) activity (Buffa, Harris et al. 2010, Ye, Hu et al. 2019) was used to calculate the HIF score by suing GSVA. GSVA is a non-parametric, unsupervised method for estimating variation of gene set enrichment through the samples of an expression data set. GSVA performs a change in coordinate systems, transforming the data from a gene by sample matrix to a gene-set by sample matrix, thereby allowing the evaluation of pathway enrichment for each sample (Hanzelmann, Castelo et al. 2013).

12. High-performance liquid chromatography (HPLC) assay

Chromatography analysis for the identification of gallic acid in LSW was conducted on a Shimadzu® HPLC system (LC-20 AT, SHIMADZU, Japan) equipped with C18 column (Shim-pack GIS: 5 µm particle size; 4.6 × 250 mm2, P/N: 227-30106-08). LSW samples were diluted with 50% methanol at a concentration of 20 mg/mL. The chromatographic separation was carried out using a mobile phase with phosphoric acid: water 0.1% (pH 3.0) as solvent A and methanol as solvent B at a flow rate of 1

mL/min. An isocratic system was used as follows: 40% A/60% B, 20 minutes. Peaks were detected at 271 nm using an UV-Vis detector (SPD-20A), and the peak for gallic acid was identified by comparing the retention time with its standard. The concentration of gallic acid standard used in this analysis is 1.0 μg/ml. The concentration of the sample is determined according to the following formula (Kupiec 2004):

$$Conc._{sample} = \frac{Area_{sample}}{Area_{standard}} * Conc._{standard}$$

13. Statistical analysis

Statistical analyses were performed in GraphPad Prism v7.02 (GraphPad Software Inc, San Diego, CA) unless otherwise indicated. No data were excluded from the studies and for all experiments, all attempts at replication were successful. For each experiment, sample size reflects the number of independent biological replicates and is provided in the figure legend. Normality of distribution was assessed using the D'Agostino-Pearson normality test. Statistical analyses of single comparisons of two groups utilized Student's *t*-test or Mann-Whitney *U*-test for parametric and non-parametric data respectively. Where appropriate, individual t-test results were corrected for multiple comparisons using the Holm-Sidak method. For multiple comparisons, one-way or two-way analysis of variance (ANOVA) with Dunnett's multiple comparison test or Kruskal-Wallis analysis with Dunn's multiple comparison test were used for parametric and non-parametric data, respectively. Results were considered significant if $P < 0.05$, where *$P < 0.05$, **$P < 0.01$, ***$P < 0.001$, ****$P < 0.0001$.

Supplementary Figures

Supplementary Figure 1. Global transcriptomic changes in MDA-MB-468 cells exposed to bioactive extracts from *Limonium Sinense*. MDA-MB-468 cells were treated with or without *Limonium Sinense* water extracts (LSW) for 48 hours followed by RNA-Seq. (**A**) Volcano plot showing up and down-regulated genes in LSW-treated MDA-MB-468 cells. Log_2FoldChange in *x*-axis and -$Log_{10}(P)$ in *y*-axis. Orange indicates up-regulation and blue down-regulation. ns: not significant. (**B**) Heatmap showing differentially expressed genes (DEGs) in LSW-treated MDA-MB-468 cells. Genes with *P*-value less than 0.05 and $|Log_2FoldChange|$ above 1 were considered as DEGs.

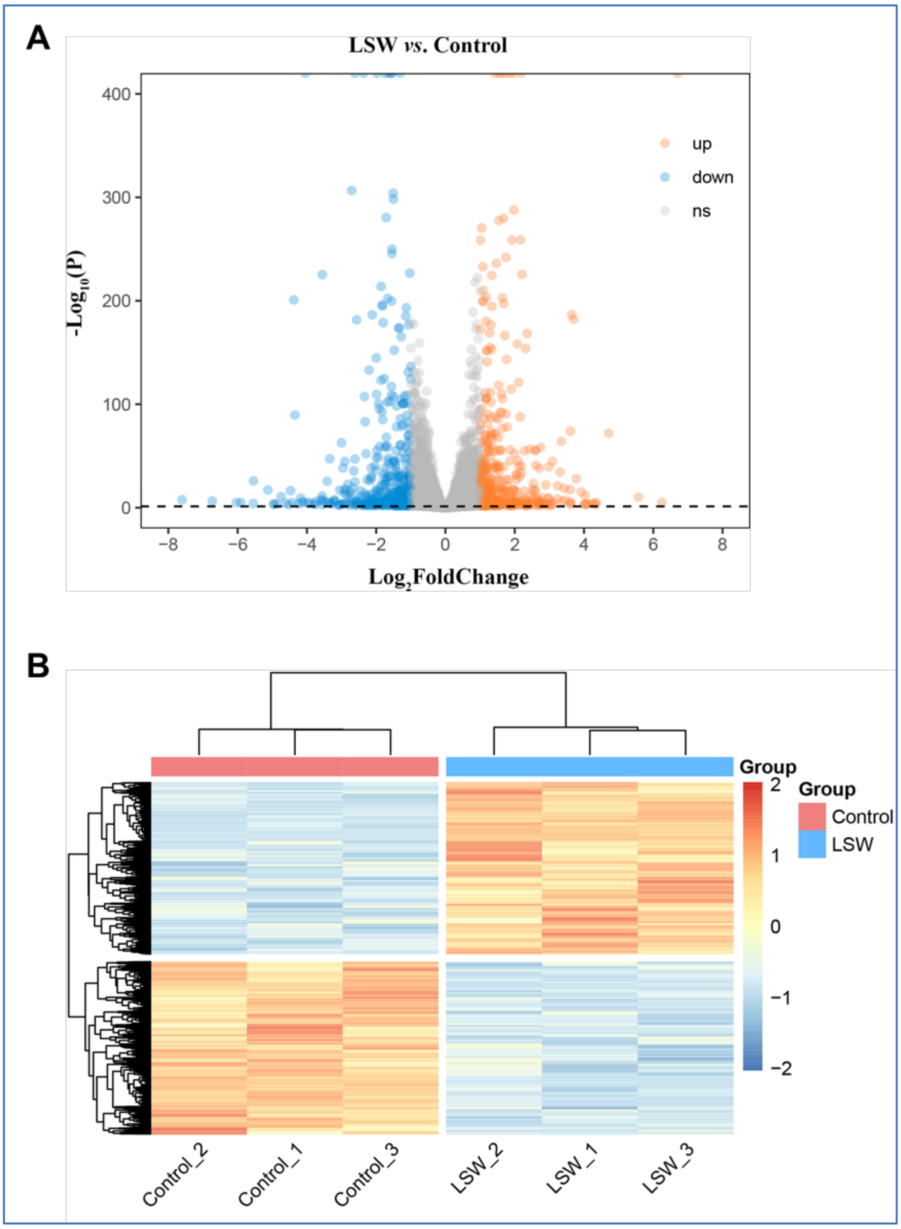

Supplementary Figure 2. Effects of bioactive extracts from *Limonium Sinense* treatment on angiogenesis. (**A**) Gene Set Enrichment analysis (GSEA) plot showing an enrichment of Hallmark_Angiogenesis in MDA-MB-468 cells treated with *Limonium Sinense* water extracts (LSW, 500 μg/ml). Normalized enrichment score (NES) and false discovery rate (FDR) are indicated. (**B**) Heatmap showing the expression of angiogenesis-related genes identified from GSEA. Red indicates up-regulation and blue down-regulation.

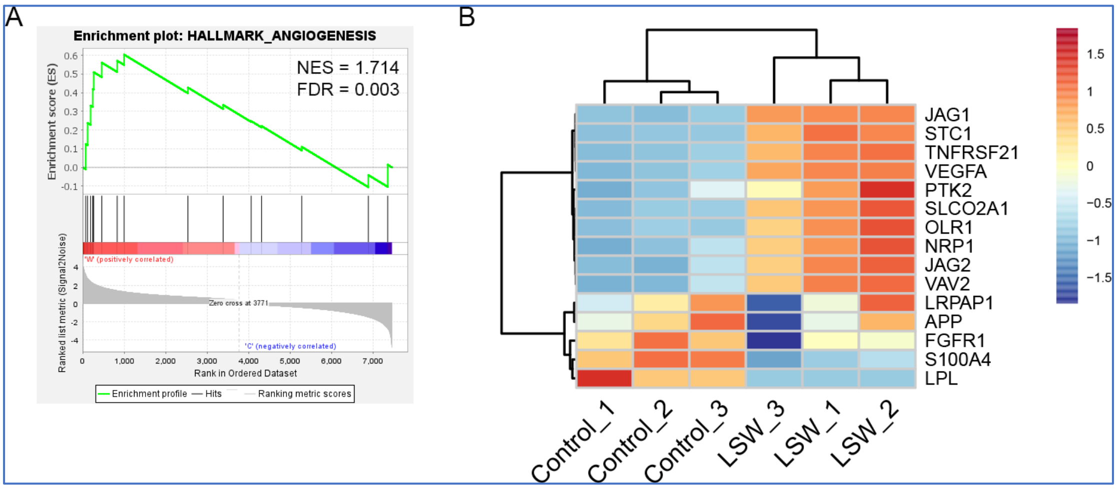

Supplementary Figure 3. Connectivity Map (CMap) analysis in MDA-MB-468 cells exposed to bioactive extracts from *Limonium Sinense*. (**A**) Heatmap showing each compound from the CMap that share Mechanism of actions (rows). Sorted by descending number of compound with shared mechanism of actions. (**B**) Heatmap showing each compound from the CMap that share gene targets in (rows). Sorted by descending number of targets. Red indicates "positive connectivity" compounds and blue "negative connectivity" compounds.

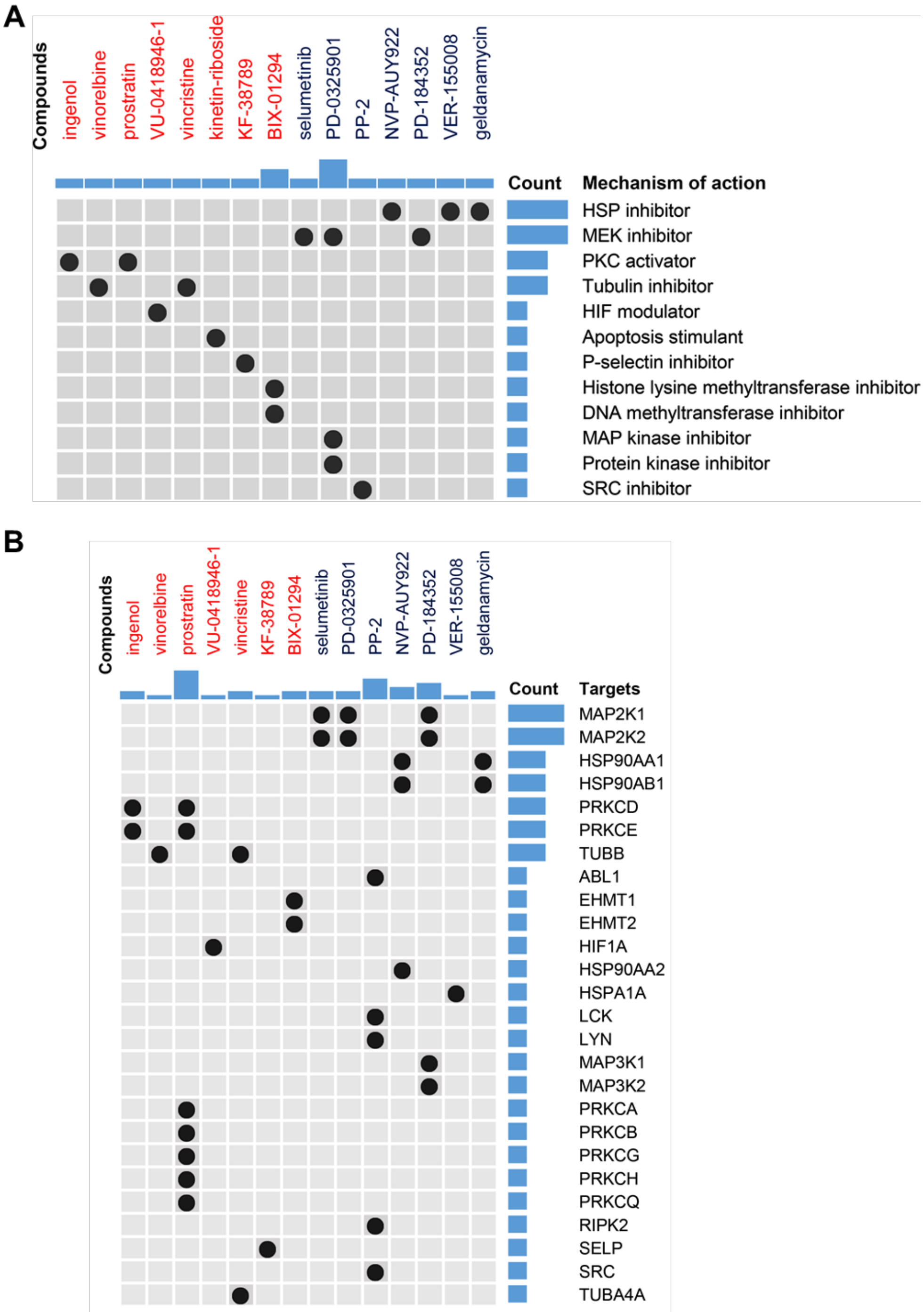

Supplementary Figure 4. Effects of bioactive extracts from *Limonium Sinense* on hypoxia-inducible factor (HIF) activation. Protein expressions of HIF-1α in HEK293T cells treated with *Limonium Sinense* water extracts (LSW, 500 μg/ml) for the indicated time. β-actin was used as a loading control.

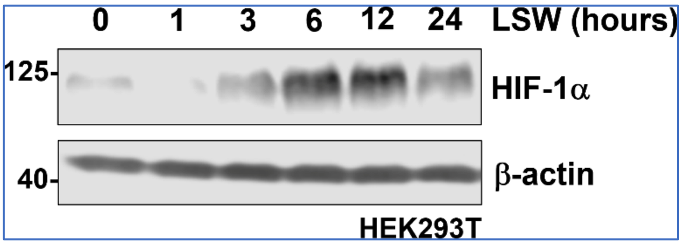

Supplementary Figure 5. Effects of bioactive extracts from *Limonium Sinense* treatment on the mRNA levels of *HIF1A* and *EGLN*1-3. Graph showing changes in the RNA-seq counts of *HIF-1A*, *EGLN1*, *EGLN2* and *EGLN3* in MDA-MB-468 cells following *Limonium Sinense* water extracts (LSW) treatment. Data are mean ± SEM; n = 3 samples per group. *$P < 0.05$; ****$P < 0.0001$ by the 2way ANOVA. ns: not significant.

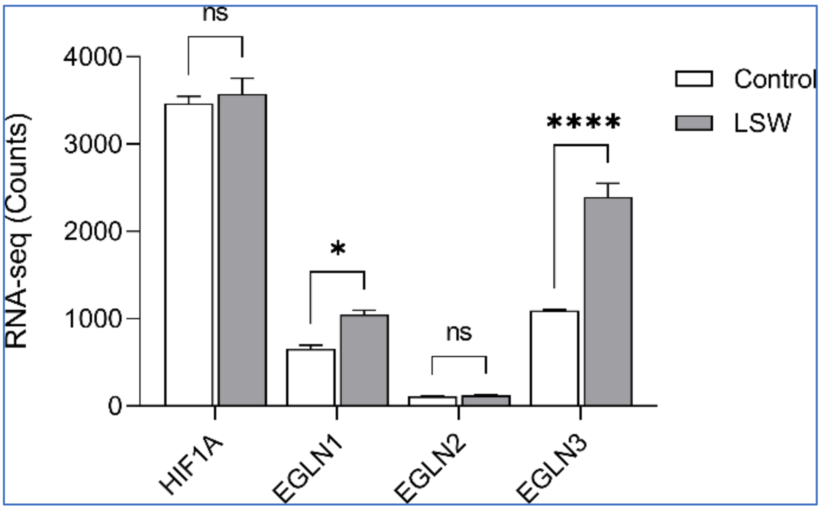

Supplementary Figure 6. Integrated analysis suggests a role for gallic acid within *Limonium Sinense* in mediating HIF activation. Flow chart showing data collections. Details are provided in the Methods.

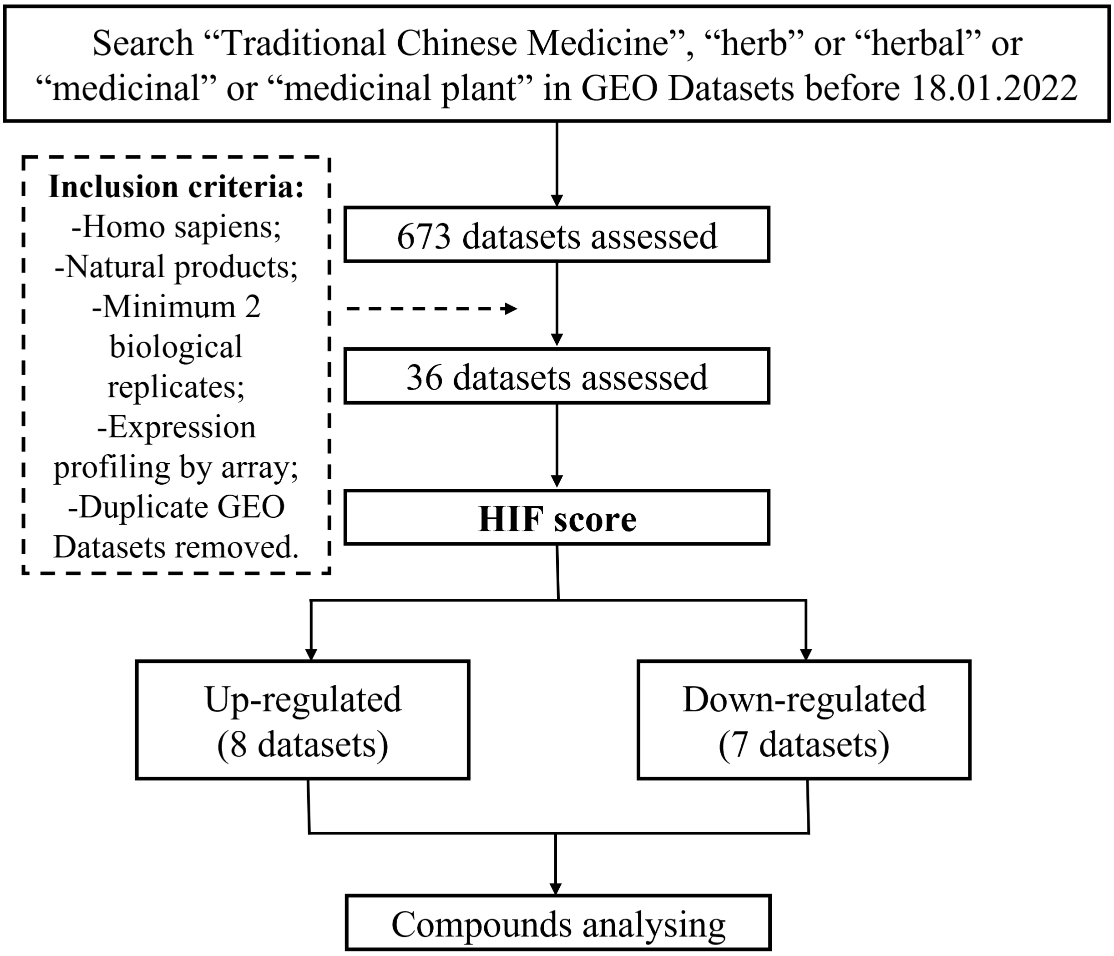

Supplementary Figure 7. Scatter plot showing natural compounds or herbal extracts that can down-regulate the HIF score. Compounds with red colour are water soluble. The sizes of circles represent the -Log10 of the *P*-values, and the colours of circles represent the HIF score mean difference of each compound compared with control samples.

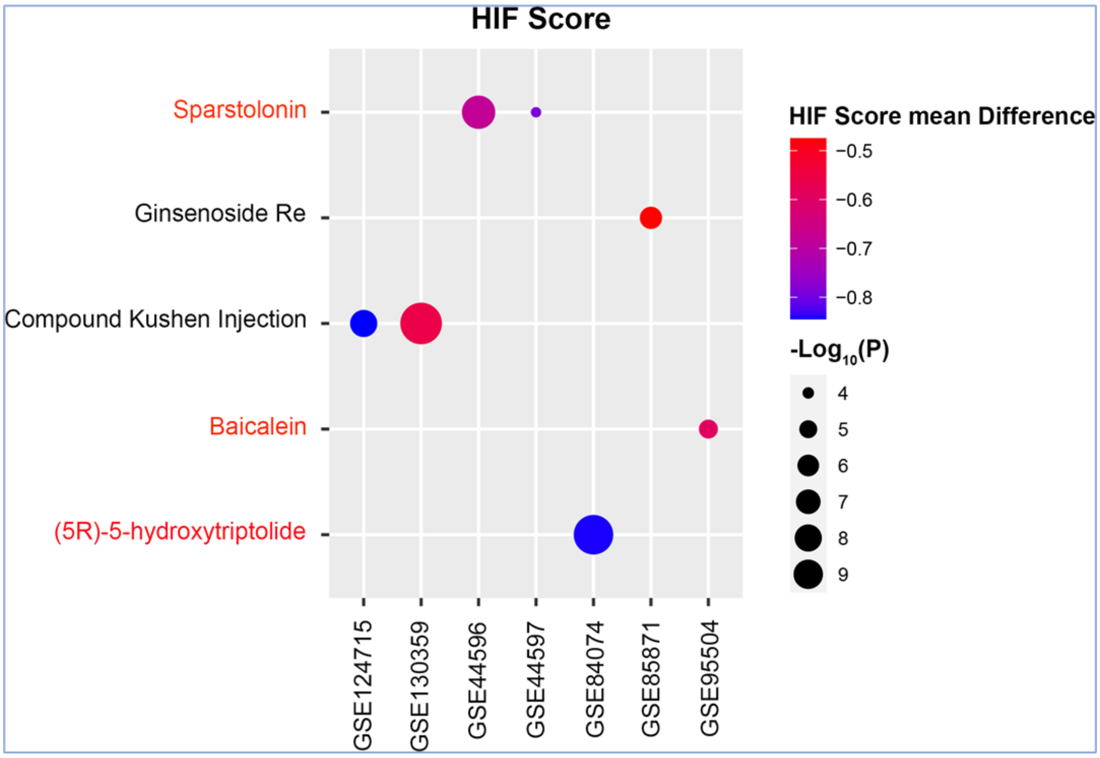

Supplementary Tables

Table S1. DEGs in MDA-MB-468 cells exposed to LSW.

Table S2. GSEA in MDA-MB-468 cells exposed to LSW.

Table S3. KEGG pathway analysis in MDA-MB-468 cells exposed to LSW.

Table S4. GO terms enrichment analysis in MDA-MB-468 cells exposed to LSW.

Table S5. CMap analysis in MDA-MB-468 cells exposed to LSW.

Table S6. Details of the GEO datasets collected for the integrated analysis.

Table S7. List of compounds that can significantly alter the HIF score.

Table rawdata_1. Effect of LSW on different types of breast cell lines

Table rawdata_2. Effect of LSW on MDA-MB-468 cells in 3D culture

Table rawdata_3. Up and down regulated genes for CMap

Table rawdata_4. Effect of LSW on cell cycle in MDA-MB-468 cells

Table rawdata_5. Effect of LSW on luciferase activity

Table rawdata_6. Expression changes of HIF target genes exposed to LSW

Table rawdata_7. LSW_vs_Control_DESeq2.csv

Table rawdata_8. GSEA_Hallmarks.csv

Table rawdata_9. go_kegg.csv

Table rawdata_10. herb_upregulated_hif_score.csv

Table rawdata_11. herb_downregulated_hif_score.csv

Table rawdata_12. LSW_DEGs.csv

R Scripts

Raw data were imported into RStudio (version 4.2.0), Microsoft Windows (version 10) and R scripts were run.
Set the working directory before run
setwd("C:/Users/hz2u19/Downloads")

1. R codes for DEG analysis in GSE205252

```
library(DESeq2) #version 1.26.0
library(apeglm)

#Download the rawdata from GSE205252
#https://www.ncbi.nlm.nih.gov/geo/query/acc.cgi?acc=GSE205252

> RNA_seq <- read.table('GSE205252_rawcount.txt', header = T, sep = '\t')
> expr = RNA_seq[, c(1, 7:12)]
> colnames(expr) <- c("Gene_ID", 'C1', 'C2', 'C3', 'W1', 'W2', 'W3')
> expr <- expr[c(2:67152), ] #remove the title of the table

#Prepare the row annotation from
#https://github.com/theislab/scvelo_notebooks/blob/master/data/biomart/mart_export
#_human.txt.

> annotation <- read.table('mart_export_human.txt', header = T, sep = '\t')
> colnames(annotation) <- c("Gene_ID", "Gene_name")
> expr <- merge(expr, annotation, by='Gene_ID')
> expr <- expr[ , c(8, 2:7)]

#Remove the duplicated rows

> expr1 <- aggregate(x=expr, by=list(expr$Gene_name), FUN=median)
> rownames(expr1) <- expr1$Group.1
> expr1 <- expr1[ , -1]
> expr1 <- na.omit(expr1) #Remove NA rows
> write.csv(expr1, "rsedata.csv")
> rsedata <- read.csv("rsedata.csv", row.names = 1)
> condition_rna_W <- factor(c(rep('C', length(rsedata[1,])/2), rep('W',
length(rsedata[1,])/2)))
> condition_rna_W
> coldata_W <- data.frame(row.names=colnames(rsedata), condition_rna_W)
```

```
> coldata_W
> dds_W <- DESeqDataSetFromMatrix(countData=rsedata, colData=coldata_W,
design=~condition_rna_W)
> nrow(dds_W)
> dds_W <- dds_W[rowSums(counts(dds_W))>10, ]
> dds_W <- DESeq(dds_W)
> resultsNames(dds_W)
> res <- lfcShrink(dds_W, coef="condition_rna_W_W_vs_C", type="apeglm")
> res <- as.data.frame(res)
> raw_count <- counts(dds_W, normalized=F)
> raw_count <- as.data.frame(raw_count)
> normalizedCounts2 <- counts(dds_W, normalized=T)
> res <- data.frame(res, normalizedCounts2)
> res <- data.frame(res,raw_count)
> write.csv(res, "LSW_vs_Control_DESeq2.csv")
```

2. R codes for Figure 2B

```
#The data was shown in Supplementary Materials: Table rawdata8. GSEA_Hallmarks.csv
library(ggplot2)
library(RColorBrewer)
wgsea<- read.csv("GSEA_Hallmarks.csv", header = TRUE)
ggplot(wgsea, aes(x = NES, y = reorder(ï..Hallmarks, -FDR),
                  color = -log10(FDR),
                  size = Gene_Count))+
                  geom_point()+
                  scale_color_gradient(low = "blue", high = "red")+
                  theme(plot.subtitle = element_text(size=11, angle=0))+
                  theme(axis.title.y = element_text(size=11, angle=0)) +
                  theme(axis.title.x = element_text(size=11, angle=0)) +
                  theme(legend.position="top") +
                  theme_bw()+
                  theme(panel.grid.major = element_line(color = "grey80",
                  size = 0.05, linetype = 1)) +
                  labs(x="Normalized Enrichment Score", y="",
                       title="GSEA:Hallmark pathways")
```

3. R codes for Figure 2C

```
#The data was shown in Supplementary Materials: Table rawdata9. go_kegg.csv
library(ggplot2)
library(RColorBrewer)
library(dplyr)
library(gridExtra)
w_go_kegg<- read.csv("go_kegg.csv", header = T)
ggplot(w_go_kegg, aes(x = Fold.Enrichment, y = reorder(Term, -PValue),
                      color = -log10(PValue), size = Gene_Count))+
                      geom_point()+
                      scale_color_gradient(low = "blue", high = "red")+
                      theme(plot.subtitle = element_text(size=11, angle=0))+
                      theme(axis.title.y = element_text(size=11, angle=0)) +
                      theme(axis.title.x = element_text(size=11, angle=0)) +
                      theme(legend.position="top") +
                      theme_bw()+
                      theme(panel.grid.major = element_line(color = "grey80",
                      size = 0.05, linetype = 1)) +
                      labs(x="Fold Enrichment", y="", title="") +
                      facet_grid(vars(ï..Category), vars(group), scales = "free_y",
                                 space = "free_y")
```

4. R codes for Figure 5B

```
#The data was shown in Supplementary Materials: Table rawdata7. LSW_vs_Control_DESeq2.csv
library(GSVA)
gsva_data<- read.csv("LSW_vs_Control_DESeq2.csv", row.names = 1)
h<-list(c('VEGFA', 'SLC2A1', 'PGAM1', 'ENO1', 'LDHA', 'TPI1', 'P4HA1', 'MRPS17',
          'CDKN3', 'ADM', 'NDRG1', 'TUBB6', 'ALDOA', 'MIF', 'ACOT7'))
gsva_w <- gsva(as.matrix(gsva_data), h, method='gsva')
write.csv(gsva_w, "lsw_hif_score.csv")
```

5. R codes for Figure 5C

#The data was shown in Supplementary Materials: Table rawdata7. LSW_vs_Control_DESeq2.csv

```
library(pheatmap)
library(RColorBrewer)
hif_genes <- read.csv("LSW_vs_Control_DESeq2.csv", row.names = 1)
hif_genes<- hif_genes[, c(6:11)]
pheatmap(hif_genes[c('VEGFA', 'SLC2A1', 'PGAM1', 'ENO1', 'LDHA',
                     'TPI1', 'P4HA1', 'MRPS17', 'CDKN3', 'ADM',
                     'NDRG1', 'TUBB6', 'ALDOA', 'MIF', 'ACOT7'),],
         scale = 'row',
         treeheight_row = 40,
         treeheight_col = 40,
         annotation_row = NA,
         annotation_names_row = FALSE,
         cluster_rows = T,
         cluster_cols = T,
         color = colorRampPalette(c("blue","white", "red"))(500),
         border_color = "grey90",
         angle_col = 45,
         fontsize_col = 10)
```

6. R codes for Figure 6A

#The data was shown in Supplementary Materials: Table rawdata10. herb_upregulated_hif_score.csv

```
library(ggplot2)
library(RColorBrewer)
herb_up_hif<- read.csv("herb_upregulated_hif_score.csv", header = T)
ggplot(herb_up_hif, aes(x = GEO.dataset, y = ï..Name,
                        color = HIF.score.mean.difference,
                        size = -log(Pvalue)))+
                        geom_point()+
                        scale_color_gradient(low = "blue", high = "red")+
                        theme(axis.text.x = element_text(size = 10, angle=90,
                              hjust = 1), axis.title.y = element_text(size=10))+
                        labs(x=" ", y=" ", title="HIF score")
```

7. R codes for Figure 6B

```
library(GEOquery)

library(limma)

library(dplyr)

library(GSVA)

gse85871_rawdata<- getGEO("GSE85871", GSEMatrix = TRUE, AnnotGPL = TRUE)

if(length(gse85871_rawdata)>1) idx<- grep("GPL570", attr(gse85871_rawdata, "names")) else idx<- 1

gse85871_rawdata<- gse85871_rawdata[[idx]]

gse85871_fulldata<- cbind(fData(gse85871_rawdata), exprs(gse85871_rawdata))

gse85871_fulldata<- subset(gse85871_fulldata, select=c(1,3,22:233))

gse85871_fulldata<-aggregate(x=gsegse85871_fulldata[,2:(ncol(gse85871_fulldata))], by=list(gse85871_fulldata$`Gene symbol`), FUN = median)

f1<- function(x){

  x<- (strsplit(x, "[///]")[[1]][1])

}

gse85871_fulldata$Group.1<- as.character(lapply(gse85871_fulldata$Group.1,f1))

gse85871_fulldata<-aggregate(x=gse85871_fulldata[,1:(ncol(gse85871_fulldata))], by=list(gse85871_fulldata$Group.1), FUN = median)

gse85871_fulldata<- gse85871_fulldata[,c(1,4:215)]

rownames(gse85871_fulldata)<- gse85871_fulldata$Group.1

gse85871_fulldata<- gse85871_fulldata[,-1]

hist(as.matrix(gse85871_fulldata))

h<-list(c('VEGFA','SLC2A1','PGAM1','ENO1','LDHA','TPI1','P4HA1',

          'MRPS17','CDKN3','ADM','NDRG1','TUBB6','ALDOA','MIF','ACOT7'))

gsva_gse85871 <- gsva(as.matrix(gse85871_fulldata), h,method='gsva')

write.csv(gsva_gse85871, "GSE85871_hif_score.csv")
```

8. R codes for Figure 6C

#Download the rawdata from GSE158788
#https://www.ncbi.nlm.nih.gov/geo/query/acc.cgi?acc=GSE158788:GSE158788_mRNA_FPKM.txt.gz
#Untar the file and transform the data into .csv file, save it as #"GSE158788_fulldata.csv".

```
library(GSVA)

gse158788 <- read.csv("GSE158788_fulldata.csv", header = T)

gse158788<- gse158788[rowSums(gse158788[, -1])>0, ] #remove all the rows value=0

gse158788<-aggregate(x=gse158788[,1:(ncol(gse158788))],
by=list(gse158788$Gene_Symbol), FUN = median)

gse158788<- gse158788[,c(1,3:17)]

rownames(gse158788)<- gse158788$Group.1

gse158788<- gse158788[,-1]

write.csv(gse158788,"GSE158788_fulldata.csv")

h<-list(c('VEGFA','SLC2A1','PGAM1','ENO1','LDHA','TPI1','P4HA1',

            'MRPS17','CDKN3','ADM','NDRG1','TUBB6','ALDOA','MIF','ACOT7'))

gsva_gse158788 <- gsva(as.matrix(gse158788), h,method='gsva')

write.csv(gsva_gse158788, "GSE158788_hif_score.csv")
```

9. R codes for Supplementary Figure 1A

```
#The data was shown in Supplementary Materials: Table rawdata7. LSW_vs_Control_DESeq2.csv
library(ggplot2)
library(RColorBrewer)
library(dplyr)
vdata<- read.csv("LSW_vs_Control_DESeq2.csv", row.names = 1)
vdata_gene_type<- vdata%>%mutate(gene_type= case_when(log2FoldChange>=1 &
                    padj<= 0.05 ~ "up", log2FoldChange<=-1 &
                    padj<= 0.05 ~ "down", TRUE ~ "ns"))
cols<- c("up"="#ff8027", "down"="#008dd9", "ns"="grey")
ggplot(data = vdata_gene_type, aes(x = log2FoldChange, y = -log10(padj))) +
            geom_point(aes(colour = gene_type), alpha=0.3, shape=16, size=1.5) +
            geom_hline(yintercept=-log10(0.05), linetype="dashed") +
            scale_colour_manual(values=cols) +
            scale_x_continuous(breaks=c(seq(-10, 10, 2)), limits = c(-8, 8)) +
            scale_y_continuous(breaks=c(seq(0, 400, 100)), limits = c(0, 400)) +
            labs(title="LSW vs Control", x="Log2FoldChange",
                    y="-Log10(padj)", colour="Expression\nchange") +
            theme_bw() +
            theme(panel.border=element_rect(colour="black", fill=NA, size=0.5),
                  panel.grid.minor=element_blank(),
                  panel.grid.major=element_blank())
```

10. R codes for Supplementary Figure 1B

```
#The data was shown in Supplementary Materials: Table rawdata12. LSW_DEGs.csv
library(pheatmap)
library(RColorBrewer)
DEG_data<- read.csv("LSW_DEGs.csv", row.names = 1)
DEG_data<- DEG_data[, c(5:10)]
col_name<- data.frame(Group=rep(c("Control", "LSW"), c(3,3)))
row.names(col_name)<- colnames(DEG_data)
my_colour = list(Group = c(Control = 'lightcoral', LSW = 'steelblue1'))
pheatmap(DEG_data, treeheight_row = 40, treeheight_col = 40,
         scale = 'row',
         annotation_col = col_name,
         annotation_colors = my_colour,
         annotation_row = NA,
         annotation_names_row = FALSE,
         clustering_distance_cols = "maximum",
         clustering_distance_rows = "manhattan",
         cluster_rows = T, cluster_cols = T,
         cutree_cols = 2, cutree_rows = 2,
         fontsize_col = 8, show_rownames = F,
         angle_col = "45")
```

11. R codes for Supplementary Figure 2B

```
#The data was shown in Supplementary Materials: Table rawdata7. LSW_vs_Control_DESeq2.csv
library(pheatmap)
library(RColorBrewer)
angio_data<- read.csv("LSW_vs_Control_DESeq2.csv", row.names = 1)
angio_data<- angio_data[, c(6:11)]
my_colour = list(Group = c(Control = 'lightcoral', LSW = 'steelblue1'))
pheatmap(angio_data[c('STC1','TNFRSF21','SLCO2A1','JAG1','VEGFA','OLR1',
                      'JAG2','NRP1','VAV2','PTK2','LRPAP1','APP',
                      'FGFR1','S100A4','LPL'),],
         treeheight_row = 20, treeheight_col = 20,
         border_color = 'grey80',
         cellheight=6, cellwidth = 18,
         scale = 'row',
         color = my_colour,
         clustering_distance_cols = "maximum",
         clustering_distance_rows = "manhattan",
         clustering_method = "ward.D",
         fontsize = 6,
         cluster_rows = T, cluster_cols = T,
         fontsize_col = 8, fontsize_row = 6,
         angle_col = "45")
```

12. R codes for Supplementary Figure 7

#The data was shown in Supplementary Materials: Table rawdata11. herb_downregulated_hif_score.csv

```
library(ggplot2)

library(RColorBrewer)

herb_down_hif<- read.csv("rawdata_herb_downregulated_hif_score.csv", header = T)

ggplot(herb_down_hif, aes(x = GEO.dataset, y = ï..Name,

                                   color = HIF.score.mean.difference,

                                   size = -log(Pvalue)))+

        geom_point()+

        scale_color_gradient(low = "blue", high = "red")+

        theme(axis.text.x = element_text(size = 10, angle=90, hjust = 1),

              axis.title.y = element_text(size=10))+

        labs(x=" ", y=" ", title="HIF score")

dev.off()
```

References

Brereton, C. J., L. Yao, E. R. Davies, Y. Zhou, M. Vukmirovic, J. A. Bell, S. Wang, R. A. Ridley, L. S. N. Dean, O. G. Andriotis, F. Conforti, L. Brewitz, S. Mohammed, T. Wallis, A. Tavassoli, R. M. Ewing, A. Alzetani, B. G. Marshall, S. V. Fletcher, P. J. Thurner, A. Fabre, N. Kaminski, L. Richeldi, A. Bhaskar, C. J. Schofield, M. Loxham, D. E. Davies, Y. Wang and M. G. Jones (2022). "Pseudohypoxic HIF pathway activation dysregulates collagen structure-function in human lung fibrosis." Elife **11**.

Buffa, F. M., A. L. Harris, C. M. West and C. J. Miller (2010). "Large meta-analysis of multiple cancers reveals a common, compact and highly prognostic hypoxia metagene." Br J Cancer **102**(2): 428-435.

Ertay, A., H. Liu, D. Liu, P. Peng, C. Hill, H. Xiong, D. Hancock, X. Yuan, M. R. Przewloka, M. Coldwell, M. Howell, P. Skipp, R. M. Ewing, J. Downward and Y. Wang (2020). "WDHD1 is essential for the survival of PTEN-inactive triple-negative breast cancer." Cell Death Dis **11**(11): 1001.

Hanzelmann, S., R. Castelo and J. Guinney (2013). "GSVA: gene set variation analysis for microarray and RNA-seq data." BMC Bioinformatics **14**: 7.

Hill, C., J. Li, D. Liu, F. Conforti, C. J. Brereton, L. Yao, Y. Zhou, A. Alzetani, S. J. Chee, B. G. Marshall, S. V. Fletcher, D. Hancock, C. H. Ottensmeier, A. J. Steele, J. Downward, L. Richeldi, X. Lu, D. E. Davies, M. G. Jones and Y. Wang (2019). "Autophagy inhibition-mediated epithelial-mesenchymal transition augments local myofibroblast differentiation in pulmonary fibrosis." Cell Death Dis **10**(8): 591.

Kupiec, T. P. (2004). "Quality-control analytical methods: high-performance liquid chromatography." Int J Pharm Compd **8**(3): 223-227.

Liu, H., A. Ertay, P. Peng, J. Li, D. Liu, H. Xiong, Y. Zou, H. Qiu, D. Hancock, X. Yuan, W. C. Huang, R. M. Ewing, J. Downward and Y. Wang (2019). "SGLT1 is required for the survival of triple-negative breast cancer cells via potentiation of EGFR activity." Mol Oncol **13**(9): 1874-1886.

Mootha, V. K., C. M. Lindgren, K. F. Eriksson, A. Subramanian, S. Sihag, J. Lehar, P. Puigserver, E. Carlsson, M. Ridderstrale, E. Laurila, N. Houstis, M. J. Daly, N. Patterson, J. P. Mesirov, T. R. Golub, P. Tamayo, B. Spiegelman, E. S. Lander, J. N. Hirschhorn, D. Altshuler and L. C. Groop (2003). "PGC-1alpha-responsive genes involved in oxidative phosphorylation are coordinately downregulated in human diabetes." Nat Genet **34**(3): 267-273.

Subramanian, A., P. Tamayo, V. K. Mootha, S. Mukherjee, B. L. Ebert, M. A. Gillette, A. Paulovich, S. L. Pomeroy, T. R. Golub, E. S. Lander and J. P. Mesirov (2005). "Gene set enrichment analysis: a knowledge-based approach for interpreting genome-wide expression profiles." Proc Natl Acad Sci U S A **102**(43): 15545-15550.

Wang, Y., F. Bu, C. Royer, S. Serres, J. R. Larkin, M. S. Soto, N. R. Sibson, V. Salter, F. Fritzsche, C. Turnquist, S. Koch, J. Zak, S. Zhong, G. Wu, A. Liang, P. A. Olofsen, H. Moch, D. C. Hancock, J. Downward, R. D. Goldin, J. Zhao, X. Tong, Y. Guo and X. Lu (2014).

"ASPP2 controls epithelial plasticity and inhibits metastasis through beta-catenin-dependent regulation of ZEB1." Nat Cell Biol **16**(11): 1092-1104.

Wang, Y., H. Xiong, D. Liu, C. Hill, A. Ertay, J. Li, Y. Zou, P. Miller, E. White, J. Downward, R. D. Goldin, X. Yuan and X. Lu (2019). "Autophagy inhibition specifically promotes epithelial-mesenchymal transition and invasion in RAS-mutated cancer cells." Autophagy **15**(5): 886-899.

Weigelt, B., P. H. Warne and J. Downward (2011). "PIK3CA mutation, but not PTEN loss of function, determines the sensitivity of breast cancer cells to mTOR inhibitory drugs." Oncogene **30**(29): 3222-3233.

Yao, L., F. Conforti, C. Hill, J. Bell, L. Drawater, J. Li, D. Liu, H. Xiong, A. Alzetani, S. J. Chee, B. G. Marshall, S. V. Fletcher, D. Hancock, M. Coldwell, X. Yuan, C. H. Ottensmeier, J. Downward, J. E. Collins, R. M. Ewing, L. Richeldi, P. Skipp, M. G. Jones, D. E. Davies and Y. Wang (2019). "Paracrine signalling during ZEB1-mediated epithelial-mesenchymal transition augments local myofibroblast differentiation in lung fibrosis." Cell Death Differ **26**(5): 943-957.

Yao, L., Y. Zhou, J. Li, L. Wickens, F. Conforti, A. Rattu, F. M. Ibrahim, A. Alzetani, B. G. Marshall, S. V. Fletcher, D. Hancock, T. Wallis, J. Downward, R. M. Ewing, L. Richeldi, P. Skipp, D. E. Davies, M. G. Jones and Y. Wang (2021). "Bidirectional epithelial-mesenchymal crosstalk provides self-sustaining profibrotic signals in pulmonary fibrosis." J Biol Chem **297**(3): 101096.

Ye, Y., Q. Hu, H. Chen, K. Liang, Y. Yuan, Y. Xiang, H. Ruan, Z. Zhang, A. Song, H. Zhang, L. Liu, L. Diao, Y. Lou, B. Zhou, L. Wang, S. Zhou, J. Gao, E. Jonasch, S. H. Lin, Y. Xia, C. Lin, L. Yang, G. B. Mills, H. Liang and L. Han (2019). "Characterization of Hypoxia-associated Molecular Features to Aid Hypoxia-Targeted Therapy." Nat Metab **1**(4): 431-444.

Raw data for 3D culture

Control

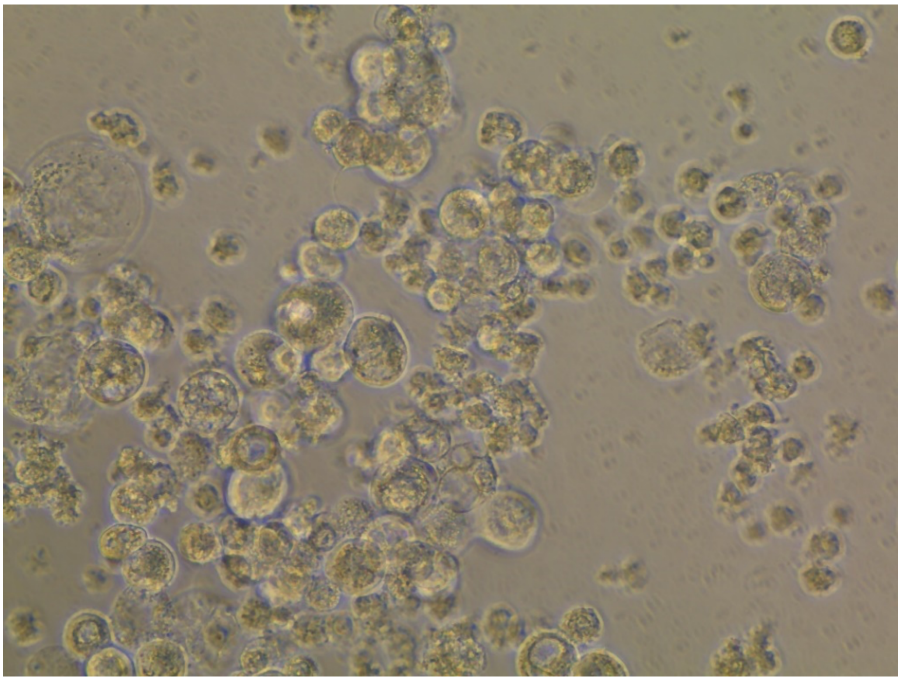

LSW treated

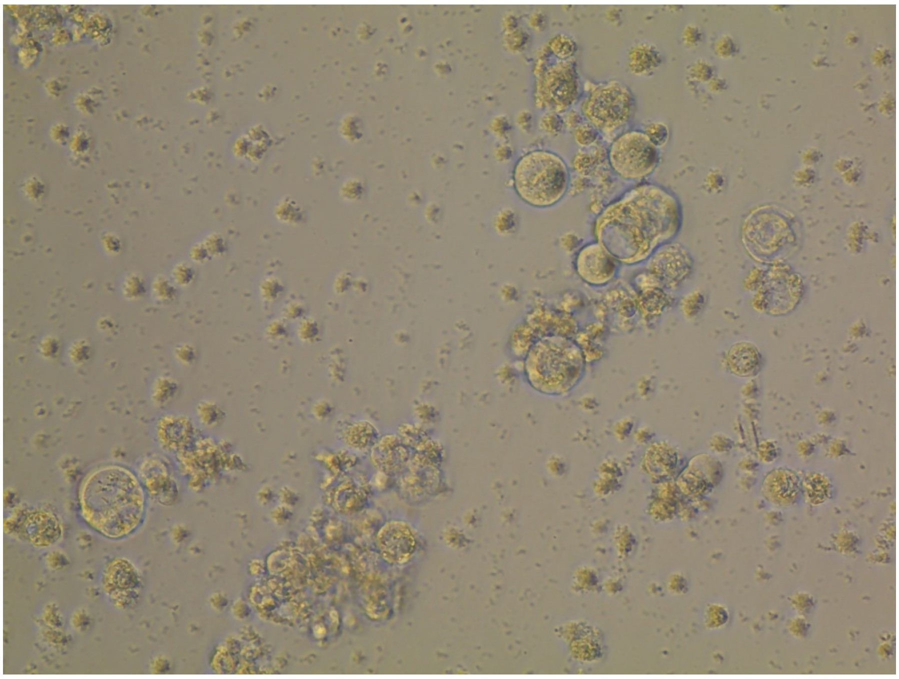

Raw data for cell cycle

Control

4/4/2021 **Guava Cell Cycle** **Version 1.3** 8:46 PM

Acquisition Date and Time: 04-APR-2021 20:22:59
Instrument Serial Number: 8472060219
Filename: C:\Users\User\Documents\Hualong\468-cc.CCY.FCS
Sample: #0001
Sample ID: 468-c-0404
User Login Name: DESKTOP-9MNKV60\User

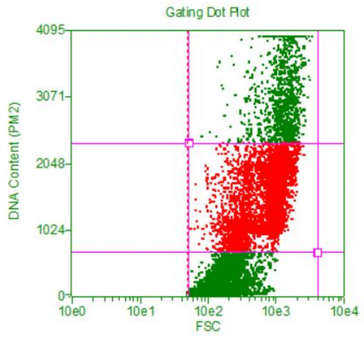

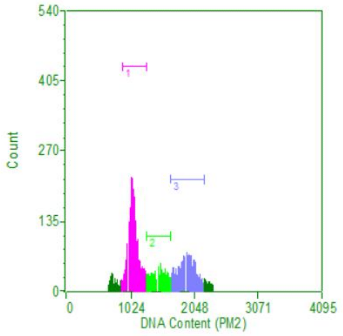

DNA Histogram Results : **Dot Plot Gated**

	M 1	M 2	M 3	M 4
**%Positive**	43.5	16.6	30.4	0.0
**PM2 Mean:**	1078	1477	1927	0
**PM2 Median:**	1068	1484	1931	0
**PM2 %CV**	7.4	7.4	7.0	0.0
**Count**	2341	896	1637	0

Particle Count

Total Particles Count : 18363
Total Particles per µL : 3232.0
Gated Particles Count : 5733
Gated Particles per µL : 1009.0

Flow Information

Flowrate : 0.12 µL/s
Volume : 5.68 µL
Duration : 00:48 m:s

Personal Cell Analysis - Guava Technologies Inc.

LSW-treated

4/4/2021	Guava Cell Cycle Version 1.3	8:43 PM

Acquisition Date and Time: 04-APR-2021 20:26:41
Instrument Serial Number: 8472060219
Filename: C:\Users\User\Documents\Hualong\468-cc.CCY.FCS
Sample: #0002
Sample ID: 468-w-0404
User Login Name: DESKTOP-9MNKV60\User

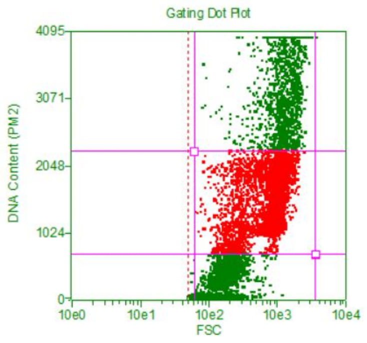

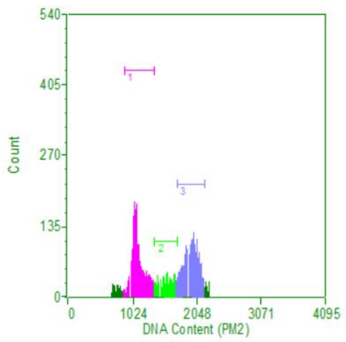

DNA Histogram Results : **Dot Plot Gated**

	M 1	M 2	M 3	M 4
%Positive	38.1	15.2	39.3	0.0
PM2 Mean:	1122	1564	1963	0
PM2 Median:	1094	1568	1970	0
PM2 %CV	9.4	6.6	5.4	0.0
Count	2004	797	2064	0

Particle Count

Total Particles Count : 18291
Total Particles per µL : 2023.2
Gated Particles Count : 5000
Gated Particles per µL : 553.1

Flow Information

Flowrate : 0.12 µL/s
Volume : 9.04 µL
Duration : 01:16 m:s

Personal Cell Analysis - Guava Technologies Inc.

Raw data for western blot

Figure 5D

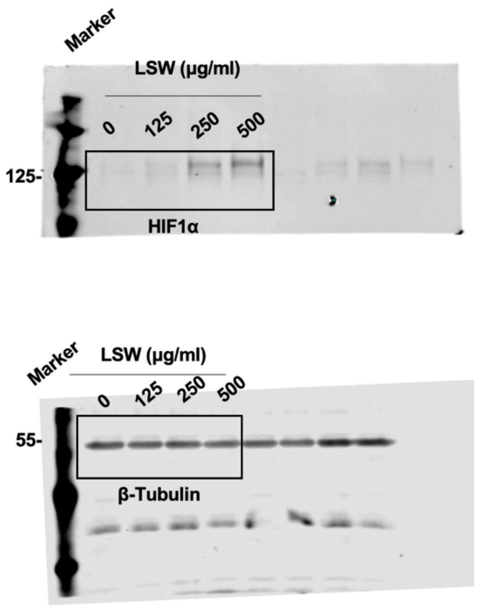

Figure 5E

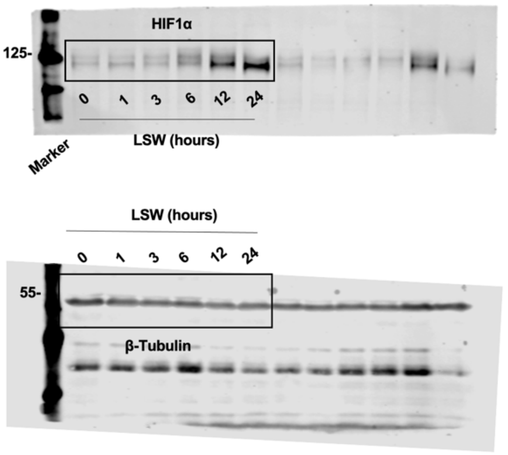

Figure 6E

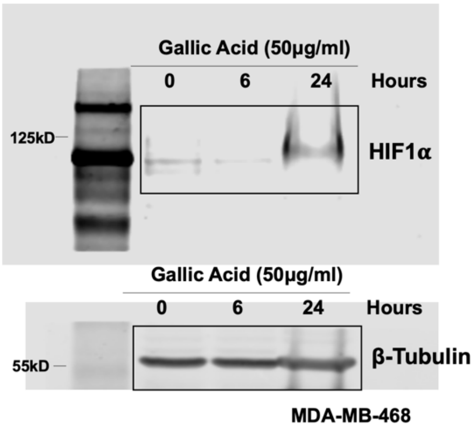

Supplementary Figure 4

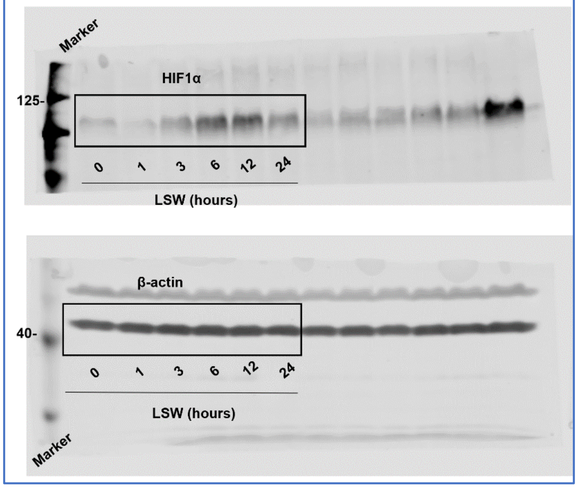

Raw data for immunofluorescence assay

Control for HIF-1α

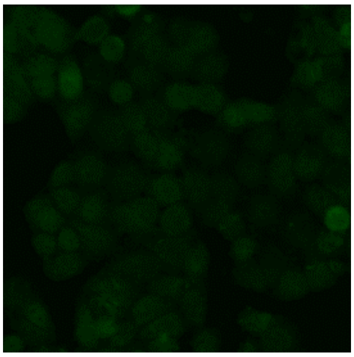

LSW-treated for HIF-1α

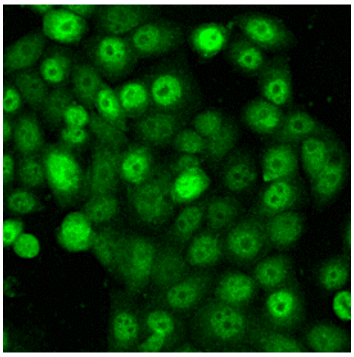

Control for DAPI

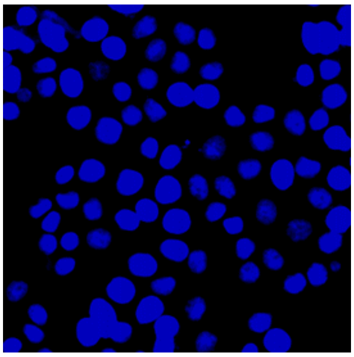

LSW-treated for DAPI

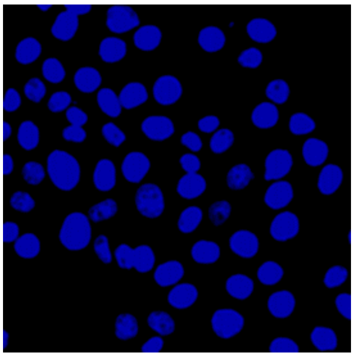

Control Merged

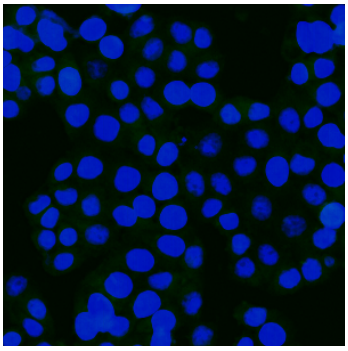

Control Merged

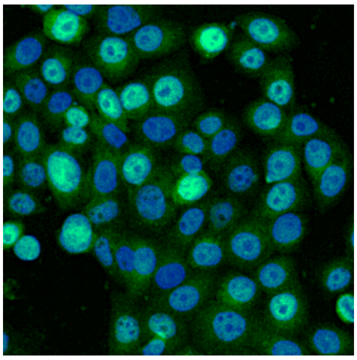

Raw data for HPLC

Gallic acid standard

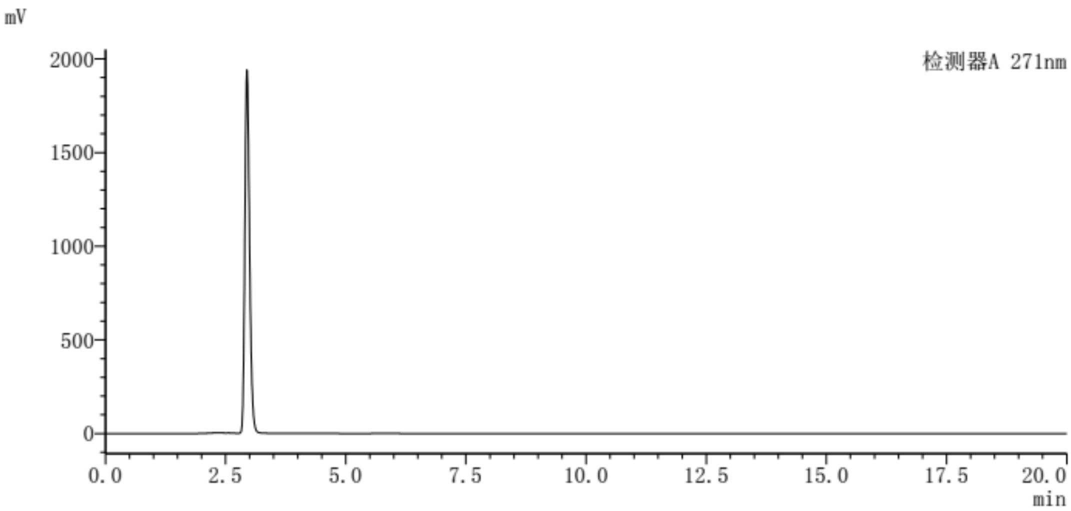

〈峰表〉

检测器A 271nm

峰号	保留时间	面积	高度	浓度	浓度单位	标记	化合物名
1	2.326	80901	3457	0.000			
2	2.563	36302	3111	0.000		V	
3	2.945	13332718	1941518	0.000		SV	
4	3.589	2147	247	0.000		T	
5	3.915	4103	466	0.000		T	
6	4.425	1426	134	0.000		T	
7	4.714	4909	290	0.000		TV	
8	5.408	1378	120	0.000		T	
9	5.940	19447	1031	0.000		TV	
10	6.776	2421	195	0.000		T	
11	8.446	1185	100	0.000			
12	16.078	2916	133	0.000			
总计		13489852	1950802				

LSW

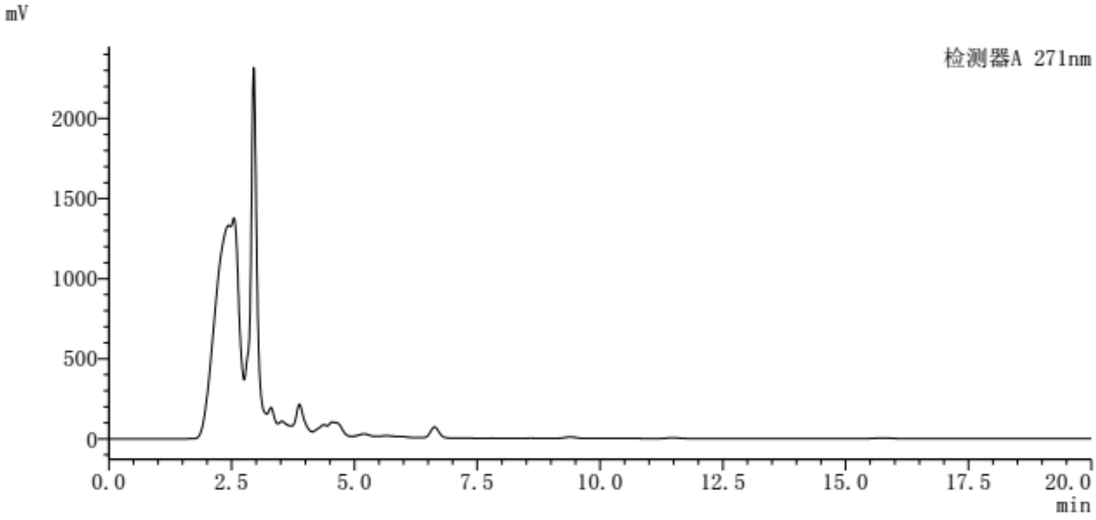

〈峰表〉

检测器A 271nm

峰号	保留时间	面积	高度	浓度	浓度单位	标记	化合物名
1	1.670	11328	2024	0.000			
2	2.437	29090519	1332127	0.000		V	
3	2.546	15374413	1377679	0.000		V	
4	2.950	29851051	2319707	0.000		SV	
5	3.301	370083	64082	0.000		T	
6	3.522	171857	21206	0.000		T	
7	3.881	1487437	152749	0.000		TV	
8	4.376	553295	52281	0.000		T	
9	4.551	1304684	75002	0.000		TV	
10	5.197	241731	16701	0.000		T	
11	5.661	144479	7809	0.000		TV	
12	5.905	54671	5268	0.000		TV	
13	6.270	4398	538	0.000		T	
14	6.630	808375	67795	0.000		TV	
15	7.317	2351	173	0.000		T	
16	7.636	3687	405	0.000		TV	
17	7.789	8391	605	0.000		TV	
18	8.206	1591	164	0.000		T	
19	8.587	18962	1390	0.000		TV	
20	9.397	168182	9329	0.000		T	
21	9.903	8449	765	0.000		TV	
22	10.022	2758	874	0.000		TV	

峰号	保留时间	面积	高度	浓度	浓度单位	标记	化合物名
23	10.237	7604	569	0.000		TV	
24	10.640	31693	1582	0.000		TV	
25	11.477	108454	5483	0.000		T	
26	14.992	2111	104	0.000			
27	15.770	117845	4667	0.000		V	
总计		79950397	5521078				
