# Supplementary material for: Integrated analysis reveals effects of bioactive ingredients from Limonium Sinense (Girard) Kuntze on hypoxia-inducible factor (HIF) activation
Source: Front Plant Sci. 2022 Oct 27;13:994036. doi: 10.3389/fpls.2022.994036 (PMC9646520; doi:10.3389/fpls.2022.994036)
Supplement: Supplementary file 1 [file DataSheet_1.zip › Additional File_submitted/Table S6.docx]

Table S6**. Details of the GEO datasets collected for the integrated analysis.**

| **Accession ID** | **Included sample information** | **Platform** |
| --- | --- | --- |
| GSE7848 [1] | Control: DMSO 6h (n=2), 24h (n=4).  *Actein*: 20 mg/ml 6h (n=2), 40 mg/ml 6h (n=2),  20 mg/ml 24h (n=3), 40 mg/ml 24h (n=3). | Affymetrix Human Genome U133A 2.0 Array |
| GSE85871 [2] | Control: DMSO 12h (n=6).  *TCM components*: 12h (n=206). |  |
| GSE23610 [3] | Control: DMSO 0.001% 6hr (n=3).  *Estradiol*: 0.1 μM 6h (n=3); *Ferulic acid*: 0.1 μM 6h (n=3), 1 μM 6h (n=3), 10 μM 6h (n=3);  *Si-Wu-Tang*: 0.0256 mg/ml 6h (n=3), 0.256 mg/ml 6h (n=3), 2.56 mg/ml 6h (n=3). | Affymetrix Human Genome U133 Plus 2.0 Array |
| GSE6800 [4] | Control: DMSO 24h (n=2).  *Cimicifuga*: 24h (n=2); *Estradiol*: 24h (n=2); *Tamoxifen*: 24h (n=2). |  |
| GSE44596 [5] | Control: DMSO 24h (n=3).  *SsnB*: 24h (n=3). |  |
| GSE44597 [5] | Control: DMSO 24h (n=2).  *SsnB*: 24h (n=2). |  |
| GSE24743 [6] | Control: DMSO 3h (n=2).  *Shikonin*: 100 nM 3h (n=2). |  |
| GSE20928 [7] | Un-treated, 12h (n=3).  *Calactin*: 0.15 mM 12h (n=3). |  |
| GSE125743 [8, 9] | Un-treated: 24h (n=3), 48h (n=6).  *CKI*: 2 mg 24h (n=3), 48h (n=6); *N_2*: 2 mg 24h (n=3), 48h (n=3); *N_3*: 2mg 24h (n=3), 48h (n=3); *OO*: 2mg 24h (n=3), 48h (n=3); *MOO*: 2 mg 24h (n=3), 48h (n=3); *N_Mac*: 2mg 48h (n=3); *N_Nme*: 2 mg 48h (n=3); *N_Omt*: 2 mg 48h (n=3); *N_Tri*: 2 mg 48h (n=3). | HiSeq X Ten |
| GSE130359 [10, 11] | *A431*: untreated_48h (n=3), vehicle_48h (n=3), *M231*: untreated_48h (n=3).  *A431*: *CKI*_48h (n=3), *Doxorubicin*_48h (n=3); *CKI*+*Doxorubicin* 48h (n=3);  *M231*: *CKI*_48h (n=3), *5-FU*_48h (n=3); *CKI*+*5FU* 48h (n=3). |  |
| GSE78512 [12] | Control: 0h (n=3), 24h (n=3), 48h (n=3).  *CKI*: 1mg 24h (n=3), 2mg 24h (n=3), 1mg 48h (n=3), 2mg 48h (n=3);  *5-FU*: 24h (n=3), 48h (n=3). | Illumina HiSeq 2500 |
| GSE116121 [13] | Control: 1h (n=3).  *F1*: 10 μM 1h (n=2);  *Rh1*: 10 μM 1h (n=2);  *VEGF*: 2.5 nM 1h (n=2);  *VEGF*+*F1*: 10 μM 1h (n=2);  *VEGF*+*Rh1*: 10 μM 1h (n=2). |  |
| GSE124715 [14] | *HEP*: untreated_0h (n=3), 24h (n=3), 48h (n=3);  *MDA*: untreated_0h (n=3), 24h (n=3), 48h (n=3).  *HEP*: VC_24h (n=3), VC_48h (n=3); *CKI*_2 mg_24h (n=3), *CKI*_2 mg_48h (n=3); *5-FU*_24h (n=3), *5-FU*_48h (n=3).  *MDA*: VC_24h (n=3), VC_48h (n=3); *CKI*_2 mg_24h (n=3), *CKI*_2 mg_48h (n=3); *5-FU*_24h (n=3), *5-FU*_48h (n=3). | Illumina NextSeq 500 |
| GSE156221 [15] | Control (n=3).  *Cotyledon orbiculata extract* (n=3). |  |
| GSE156445 [16] | Control (n=3).  *Cissampelos pareira* 1 μg (n=3), 10 μg (n=3), 100 μg (n=3), 500 μg (n=3), 1000 μg (n=3). | Affymetrix Human Transcriptome Array 2.0 |
| GSE95504 [17] | Control: DMSO (n=3).  *Baicalein*: 40 μM (n=3), 80 μM (n=3). |  |
| GSE128856 [18] | *BPH1* control (n=2), *WPMY-1* control (n=2).  *BPH1* *PAO* 250 μg/ml (n=2), *WPMY-1* *PAO* 250 μg/ml (n=2). |  |
| GSE112908 [19] | Control: DMSO 0.1% 48h (n=3).  *Oridonin*: 10 μM 48h (n=3). | Illumina HiSeq 4000 |
| GSE139929 [20] | Control (n=3).  *APBBR1*: (n=3). |  |
| GSE85746 [21] | Untreated: 0h (n=2), 6h (n=3), 12h (n=3), 18h (n=2), 24h (n=3), 48h (n=3).  *CCL*: 6h (n=3), 12h (n=3), 18h (n=3), 24h (n=2), 48h (n=2). | Affymetrix Human Gene 2.0 ST Array |
| GSE110335 [22] | Control: DMSO 6h (n=3).  *Glycyrrhetinic acid*: 40 μM 6h (n=3). |  |
| GSE81835 [23] | Untreated (n=3).  *Suduxing*: 0.001 μg/ml (n=3), 0.01 μg/ml (n=3). | Agilent-028004 SurePrint G3 Human GE 8x60K Microarray |
| GSE53415 | Control: 2h (n=3), 4h (n=3), 8h (n=3).  *Berberine*: 2h (n=3), 4h (n=3), 8h (n=3). |  |
| GSE189697 [24] | Control (n=3).  *14,15β-dihydroxyklaineanone*: 24h (n=3). | Illumina NovaSeq 6000 |
| GSE182007 | Untreated (n=3), vehicle (n=6).  *Trichostatin A*: 0.64 μM (n=3);  *Wortmannin*: 1.95 μM (n=6);  *W-BR*: 500 μg/ml (n=3), 100 μg/ml (n=3), 20 μg/ml (n=3);  *E-BR*: 133 μg/ml (n=3), 26.6 μg/ml (n=3), 5.32 μg/ml (n=3);  *Saikosaponin D*: (n=63);  *BR-Combination*: (n=9). | MGISEQ-2000RS |
| GSE164934 [25] | Control: placebo (n=13).  *Herbal preparation*: (n=13). | Affymetrix Human Gene 1.1 ST Array |
| GSE99820 [26] | Control: vehicle (n=3).  *WCE*: (n=3). | Illumina HiScanSQ |
| GSE100224 | Control: vehicle (n=2).  *WCE*: (n=2). | Illumina HumanHT-12 V4.0 expression beadchip |
| GSE86798 [27] | Control: (n=2); *IL-1β*: 1 ng/ml (n=2);  *Indomethacin*+*IL-1β*: (n=2);  *Gallic acid*+*IL-1β*: (n=6);  *Piperine*+*IL-1β*: (n=4);  *AVS023*+*IL-1β*: (n=6). |  |
| GSE61926 [28] | Control: (n=8).  *Rikkunshito*: (n=9). | Illumina humanRef-8 v2.0 expression beadchip |
| GSE42236 [29] | *SHSY5Y_*Control: (n=24); *IMR32_*Control: (n=3).  *SHSY5Y_Gelsemium*: (n=24); *IMR32*_*Gelsemium*: (n=3) | NimbleGen Homo sapiens Expression Array |
| GSE64111 [30] | Control: DMSO (n=4).  *Pachymic acid*: (n=4). | Affymetrix Human Gene Expression Array |
| GSE3983 [31] | Untreated: (n=4).  *Agaricus*: (n=4). | ABI Human Genome Survey Microarray v2.0 |
| GSE24191 [32] | Untreated: (n=8).  *Cordyceps sinensis*: (n=4);  *LSP*: (n=4);  *LSP*+*Cordyceps sinensis*: (n=4). | MBPL human 30k P7 |
| GSE84074 [33] | Control: (n=5).  *(5R)-5-hydroxytriptolide*: (n=5). | Agilent-062918 Human lncRNA array V4.0 |
| GSE103044 [34] | Control: (n=3).  *Folate deficiency*: (n=3);  *Folate repletion*: (n=3). | Illumina HiSeq 2000 |

References

1. Einbond, L.S., et al., *The growth inhibitory effect of actein on human breast cancer cells is associated with activation of stress response pathways.* Int J Cancer, 2007. **121**(9): p. 2073-83.

2. Lv, C., et al., *The gene expression profiles in response to 102 traditional Chinese medicine (TCM) components: a general template for research on TCMs.* Sci Rep, 2017. **7**(1): p. 352.

3. Wen, Z., et al., *Discovery of molecular mechanisms of traditional Chinese medicinal formula Si-Wu-Tang using gene expression microarray and connectivity map.* PLoS One, 2011. **6**(3): p. e18278.

4. Gaube, F., et al., *Gene expression profiling reveals effects of Cimicifuga racemosa (L.) NUTT. (black cohosh) on the estrogen receptor positive human breast cancer cell line MCF-7.* BMC Pharmacol, 2007. **7**: p. 11.

5. Bateman, H.R., et al., *Sparstolonin B inhibits pro-angiogenic functions and blocks cell cycle progression in endothelial cells.* PLoS One, 2013. **8**(8): p. e70500.

6. Ahmed, K., et al., *Chemical inducers of heat shock proteins derived from medicinal plants and cytoprotective genes response.* Int J Hyperthermia, 2012. **28**(1): p. 1-8.

7. Lee, C.C., et al., *The small molecule calactin induces DNA damage and apoptosis in human leukemia cells.* Eur J Cancer Prev, 2012. **21**(5): p. 467-73.

8. Nourmohammadi, S., et al., *Effect of Compound Kushen Injection, a Natural Compound Mixture, and Its Identified Chemical Components on Migration and Invasion of Colon, Brain, and Breast Cancer Cell Lines.* Front Oncol, 2019. **9**: p. 314.

9. Aung, T.N., et al., *Fractional Deletion of Compound Kushen Injection Indicates Cytokine Signaling Pathways are Critical for its Perturbation of the Cell Cycle.* Sci Rep, 2019. **9**(1): p. 14200.

10. Shen, H., et al., *Understanding the Mechanistic Contribution of Herbal Extracts in Compound Kushen Injection With Transcriptome Analysis.* Front Oncol, 2019. **9**: p. 632.

11. Shen, H., et al., *A New Strategy for Identifying Mechanisms of Drug-drug Interaction Using Transcriptome Analysis: Compound Kushen Injection as a Proof of Principle.* Sci Rep, 2019. **9**(1): p. 15889.

12. Qu, Z., et al., *Identification of candidate anti-cancer molecular mechanisms of Compound Kushen Injection using functional genomics.* Oncotarget, 2016. **7**(40): p. 66003-66019.

13. Kang, J.I., et al., *Pro-angiogenic Ginsenosides F1 and Rh1 Inhibit Vascular Leakage by Modulating NR4A1.* Sci Rep, 2019. **9**(1): p. 4502.

14. Cui, J., et al., *The effect of compound kushen injection on cancer cells: Integrated identification of candidate molecular mechanisms.* PLoS One, 2020. **15**(7): p. e0236395.

15. Makhafola, T.J., et al., *Apoptosis in Cancer Cells Is Induced by Alternative Splicing of hnRNPA2/B1 Through Splicing of Bcl-x, a Mechanism that Can Be Stimulated by an Extract of the South African Medicinal Plant, Cotyledon orbiculata.* Front Oncol, 2020. **10**: p. 547392.

16. Haider, M., et al., *Transcriptome analysis and connectivity mapping of Cissampelos pareira L. provides molecular links of ESR1 modulation to viral inhibition.* Sci Rep, 2021. **11**(1): p. 20095.

17. Bie, B., et al., *Baicalein, a Natural Anti-Cancer Compound, Alters MicroRNA Expression Profiles in Bel-7402 Human Hepatocellular Carcinoma Cells.* Cell Physiol Biochem, 2017. **41**(4): p. 1519-1531.

18. Dong, Y., et al., *Pao Pereira extract suppresses benign prostatic hyperplasia by inhibiting inflammation-associated NFkappaB signaling.* BMC Complement Med Ther, 2020. **20**(1): p. 150.

19. Zhan, Z., et al., *Oridonin alleviates hyperbilirubinemia through activating LXRalpha-UGT1A1 axis.* Pharmacol Res, 2022. **178**: p. 106188.

20. Mao, Z.J., et al., *Combined Use of Astragalus Polysaccharide and Berberine Attenuates Insulin Resistance in IR-HepG2 Cells via Regulation of the Gluconeogenesis Signaling Pathway.* Front Pharmacol, 2019. **10**: p. 1508.

21. Ng, Y.K. *The Effect of Cratoxylum Cochinchinense Lour (CCL) On Global Mrna Gene Expression In Hepg2 Liver Cancer Cells*. 2017.

22. Kuk, H., et al., *Glycyrrhetinic Acid Antagonizes Pressure-Induced Venous Remodeling in Mice.* Front Physiol, 2018. **9**: p. 320.

23. Liu, Y., et al., *Chinese herbal extract Su-duxing had potent inhibitory effects on both wild-type and entecavir-resistant hepatitis B virus (HBV) in vitro and effectively suppressed HBV replication in mouse model.* Antiviral Res, 2018. **155**: p. 39-47.

24. Shu, Y., et al., *DHOK Exerts Anti-Cancer Effect Through Autophagy Inhibition in Colorectal Cancer.* Front Cell Dev Biol, 2021. **9**: p. 760022.

25. Esser, D., et al., *Ayurvedic Herbal Preparation Supplementation Does Not Improve Metabolic Health in Impaired Glucose Tolerance Subjects; Observations from a Randomised Placebo Controlled Trial.* Nutrients, 2021. **13**(1).

26. Tsai, C.H., et al., *A standardized herbal extract mitigates tumor inflammation and augments chemotherapy effect of docetaxel in prostate cancer.* Sci Rep, 2017. **7**(1): p. 15624.

27. Thamsermsang, O., et al., *IL-1beta-induced modulation of gene expression profile in human dermal fibroblasts: the effects of Thai herbal Sahatsatara formula, piperine and gallic acid possessing antioxidant properties.* BMC Complement Altern Med, 2017. **17**(1): p. 32.

28. Chen, Y.C., et al., *Whole genome gene expression changes and hematological effects of rikkunshito in patients with advanced non-small cell lung cancer receiving first line chemotherapy.* Exp Ther Med, 2017. **14**(3): p. 2040-2052.

29. Marzotto, M., et al., *Extreme sensitivity of gene expression in human SH-SY5Y neurocytes to ultra-low doses of Gelsemium sempervirens.* BMC Complement Altern Med, 2014. **14**: p. 104.

30. Cheng, S., et al., *Pachymic acid inhibits growth and induces apoptosis of pancreatic cancer in vitro and in vivo by targeting ER stress.* PLoS One, 2015. **10**(4): p. e0122270.

31. Grinde, B., G. Hetland, and E. Johnson, *Effects on gene expression and viral load of a medicinal extract from Agaricus blazei in patients with chronic hepatitis C infection.* Int Immunopharmacol, 2006. **6**(8): p. 1311-4.

32. Li, C.Y., et al., *Gene expression profiling of dendritic cells in different physiological stages under Cordyceps sinensis treatment.* PLoS One, 2012. **7**(7): p. e40824.

33. Guo, S., et al., *(5R)-5-Hydroxytriptolide (LLDT-8) induces substantial epigenetic mediated immune response network changes in fibroblast-like synoviocytes from rheumatoid arthritis patients.* Sci Rep, 2019. **9**(1): p. 11155.

34. Savini, C., et al., *Folate Repletion after Deficiency Induces Irreversible Genomic and Transcriptional Changes in Human Papillomavirus Type 16 (HPV16)-Immortalized Human Keratinocytes.* Int J Mol Sci, 2019. **20**(5).
